# Proteogenomic analysis of acute myeloid leukemia associates relapsed disease with reprogrammed energy metabolism both in adults and children

**DOI:** 10.1038/s41375-022-01796-7

**Published:** 2022-12-26

**Authors:** Svea Stratmann, Mattias Vesterlund, Husen M. Umer, Saeed Eshtad, Aron Skaftason, Morten Krogh Herlin, Christer Sundström, Anna Eriksson, Martin Höglund, Josefine Palle, Jonas Abrahamsson, Kirsi Jahnukainen, Monica Cheng Munthe-Kaas, Bernward Zeller, Katja Pokrovskaja Tamm, Cecilia Lindskog, Lucia Cavelier, Janne Lehtiö, Linda Holmfeldt

**Affiliations:** 1 Department of Immunology, Genetics and Pathology, Science for Life Laboratory, Uppsala University, Uppsala, Sweden; 2 Department of Oncology-Pathology, Karolinska Institutet, and Science for Life Laboratory, Stockholm, Sweden; 3 Center for Hematology and Regenerative Medicine, Department of Medicine, Karolinska Institutet, Stockholm, Sweden; 4 Department of Molecular Medicine and Surgery, Karolinska Institutet, Stockholm, Sweden; 5 Department of Clinical Medicine, Aarhus University, Aarhus, Denmark; 6 Department of Pediatrics and Adolescent Medicine, Aarhus University Hospital, Aarhus, Denmark; 7 Department of Medical Sciences, Uppsala University, Uppsala, Sweden; 8 Department of Women’s and Children’s Health, Uppsala University, Uppsala, Sweden; 9 Department of Pediatrics, Institute of Clinical Sciences, Sahlgrenska Academy at University of Gothenburg, Gothenburg, Sweden; 10 Children’s Hospital, University of Helsinki and Helsinki University Central Hospital, Helsinki, Finland; 11 Norwegian Institute of Public Health, Oslo, Norway; 12 Department of Pediatric Oncology and Hematology, Oslo University Hospital, Oslo, Norway; 13 Department of Oncology and Pathology, Karolinska Institutet, Bioclinicum, Solna, Sweden; 14 The Beijer Laboratory, Uppsala, Sweden

**Keywords:** Acute myeloid leukaemia, Acute myeloid leukaemia

## Abstract

Despite improvement of current treatment strategies and novel targeted drugs, relapse and treatment resistance largely determine the outcome for acute myeloid leukemia (AML) patients. To identify the underlying molecular characteristics, numerous studies have been aimed to decipher the genomic- and transcriptomic landscape of AML. Nevertheless, further molecular changes allowing malignant cells to escape treatment remain to be elucidated. Mass spectrometry is a powerful tool enabling detailed insights into proteomic changes that could explain AML relapse and resistance. Here, we investigated AML samples from 47 adult and 22 pediatric patients at serial time-points during disease progression using mass spectrometry-based in-depth proteomics. We show that the proteomic profile at relapse is enriched for mitochondrial ribosomal proteins and subunits of the respiratory chain complex, indicative of reprogrammed energy metabolism from diagnosis to relapse. Further, higher levels of granzymes and lower levels of the anti-inflammatory protein CR1/CD35 suggest an inflammatory signature promoting disease progression. Finally, through a proteogenomic approach, we detected novel peptides, which present a promising repertoire in the search for biomarkers and tumor-specific druggable targets. Altogether, this study highlights the importance of proteomic studies in holistic approaches to improve treatment and survival of AML patients.

## Introduction

Over the lastdecades, research efforts in acute myeloid leukemia (AML) have to a great extent uncovered the inter- and intratumor heterogeneity at AML onset, resulting in improved risk classification [1, 2] and novel treatment options [3–5]. However, prognostic validation and treatment decisions in AML are mainly based on knowledge derived from genomic- and transcriptomic studies, although these changes do not always directly reflect disease pathophysiology.

Proteomics provides direct molecular phenotype characterization of AML and hence could enable better identification of potential druggable targets. High resolution isoelectric focusing liquid chromatography mass spectrometry (HiRIEF LC-MS) is an in-depth proteomic approach that allows the detection of smaller variations in protein levels and low abundant proteins through reduction in peptide complexity by fractionation [6, 7]. Additionally, through proteogenomic analyses exploiting the peptide pI information from experimental fractionation, a sensitive detection of peptides generated from genomic and transcriptomic lesions becomes possible [8]. One further advantage compared with standard genomic and transcriptomic analysis approaches, is the possibility to identify novel peptides resulting from translation of regions thought to be silent or non-coding. Exploiting these putative neoantigens offers great potential to detect novel immunotherapeutic targets.

A few studies have performed global proteomics and phosphoproteomics on AML patient material (e.g., [9–17]). The studies carried out thus far mainly served to identify diagnostic or prognostic markers, or to investigate the efficacy of novel treatments. However, current knowledge on proteomic alterations in relapsed AML remains sparse, including knowledge on the role of these alterations during leukemia progression, which commonly is associated with treatment resistance. To-date, only one published study exists including patient-matched diagnosis-relapse samples from seven AML cases [18].

To shed further light on the proteomic landscape of AML cells, we profiled 119 proteomes (diagnosis, *n* = 42; relapse [R], *n* = 71; primary resistant [PR], *n* = 6) from 47 adult and 22 pediatric R/PR AML cases in an unbiased, MS-based analysis. We report higher levels at relapse of proteins involved in regulation of mitochondrial translation and peptide biosynthesis, as well as relapse-associated pro-inflammatory proteomic signatures. In addition, several splicing-related proteins showed higher levels at relapse compared to diagnosis. We further incorporated genomic [19] and transcriptomic data [20] from patient-matched tumor cells into the analysis. By virtue of this analysis, we identified novel peptides that can be further investigated as promising AML-specific drug targets, especially regarding immunotherapeutic approaches.

## Methods

### Details of the methods are provided in the Supplementary Information

#### Patients and normal controls

The study included relapse and PR tumor samples from 47 adult and 22 pediatric AML patients, together with diagnosis samples for 42 of these patients (25 adults; 17 children; total of 119 AML samples). Inclusion criteria were AML cases with available R/PR material of sufficient quality and yield, which were accessible through various biobanks in the Nordic countries. Acute promyelocytic leukemia cases were excluded. Informed consent was obtained from all patients or their legal guardians according to the Declaration of Helsinki. The study was approved by the Uppsala Ethical Review Board (Sweden) and the Regional Ethical Committee South-East (Norway). Sixty-two of the patients had *de novo* AML, three had therapy-related AML (t-AML), two had a prior diagnosis of myelodysplastic syndromes (MDS), and two had t-MDS-AML. Event-free survival (EFS) was measured as the time from diagnosis until first relapse or initial treatment failure, with the latter indicated by time = 0. The median length of EFS for relapse cases was 10.0 months (range: 1.1–126.0) for adults and 9.9 months (range: 2.3–19.1) for children (Table 1). Detailed clinical information and sample characteristics are summarized in Supplementary Tables 1–3.

**Table 1:** Patient cohort.

Number of patients, *n* (%)			69 (100)
	Adult cases		47 (68.1)
		Elderly (≥60 y)	25 (36.2)
		Adult (40–59 y)	16 (23.2)
		Young adult (19–39 y)	6 (8.7)
	Pediatric cases		22 (31.9)
		Adolescent (15–18 y)	2 (2.9)
		Child (3–14 y)	13 (18.8)
		Infant (<3 y)	7 (10.1)
	Sex, female		37 (53.6)
	Background	de novo AML	62 (89.9)
		Potential t-AML	3 (4.3)
		MDS-AML	2 (2.9)
		t-MDS-AML	2 (2.9)
Number of tumor samples, *n* (%)			119 (100)
	Diagnosis samples		42 (35.3)
	Relapse samples		71 (59.7)
		R1 and R1-P	56 (47.1)
		R2 and R2-P	12 (10.1)
		R3	3 (2.5)
	Primary resistant samples		6 (5.0)
Mean age at onset (y)	Adult cases	59.5 (median: 62.2; range: 20.5–83.1)	
	Pediatric cases	7.7 (median: 7.3; range: 0.4–17.5)	
Mean length of EFS (m; D > R1)	Adult relapse cases	16.3 (median: 10.0; range: 1.1–126.0)	
	Pediatric relapse cases	9.9 (median: 9.9; range: 2.3–19.1)	
Mean length of OS (m)	Adult relapse cases	39.9 (median: 21.0; range: 1.5–271.1)	
	Pediatric relapse cases	52.9 (median: 18.8; range: 5.6–215.0)	
Sampling duration	1995–2016		

Detailed biological and clinical data for each patient/sample are presented in Supplementary Tables 1 and 2.

*D* Diagnosis, *EFS* event-free survival as time to first relapse, *MDS* Myelodysplastic syndromes, *OS* Overall survival, *R1/2/3* Sequential relapses, *R1/2-P* Persistent relapse specimen, *t-AML* Treatment related AML.

CD34-expressing bone marrow (BM) cells from five healthy donors (ABM017F; AllCells Inc, Alameda, CA, USA) were used as normal controls (“BM-controls”) for the proteomic and proteogenomic analyses (Supplementary Table 4).

#### Sample preparation and mass spectrometry

The peptide composition of 119 tumor samples from 69 AML patients and five BM-control samples was analyzed by HiRIEF LC-MS, performed at the Clinical Proteomics Mass Spectrometry facility at Karolinska University Hospital and Science for Life Laboratory, Sweden. In brief, cryopreserved, mononuclear cells mainly from BM or peripheral blood were isolated through Ficoll gradient centrifugation. AML samples with leukemia cell content <80% and sufficient cell count were purified by immune-based depletion of non-tumor cells (Supplementary Table 5). Nucleic acids and proteins were extracted via the AllPrep DNA/RNA/Protein Kit (Qiagen, Hilden, Germany) according to the manufacturer’s instructions. Protein pellets were dissolved in lysis buffer (4% SDS, 20 mM HEPES pH 7.6, 1 mM DTT). Protein concentrations were quantified via the BCA Protein Assay Kit with reducing agent compatibility (Thermo Fisher Scientific, Waltham, MA, USA).

The samples were prepared for MS analysis by using a modified version of the SP3 protein cleanup and digestion protocol [21]. Peptides were labeled by Isobaric Mass Tag Labeling (TMT10-plex reagent; Thermo Fisher Scientific) according to the manufacturer’s protocol, and separated by immobilized pH gradient - isoelectric focusing (IPG-IEF) on 3-10 strips as described previously [6]. Extracted peptide fractions from the IPG-IEF were separated by using an online 3000 RSLCnano system coupled to a Thermo Fisher Scientific Q Exactive-HF.

#### Protein abundance analysis

MS-spectra were matched to the human protein subset of Ensembl v.75. Absolute intensity values of the MS-spectra were converted to ratios based on an internal reference pool, and log2-transformed. The respective adult and pediatric list with quantified proteins was filtered to only retain proteins found in each adult sample, respectively, in each pediatric sample within the cohort (Supplementary Table 6). Subsequently, Qlucore omics explorer v.3.6 was utilized for protein abundance analysis and underlying calculations.

#### Immunoblotting

At least 36 μg total lysate was separated via SDS-PAGE, followed by transfer to nitrocellulose membranes and antibody-hybridization towards MTIF3, NDUFC2, and β-Actin (Supplementary Table 5).

#### Proteogenomic analysis

HiRIEF LC-MS-derived proteomics spectra data were searched against a fractionated peptide database generated from the three-frame translation of transcript sequences. The transcripts were assembled based on transcriptomic (RNA-seq) data from the same AML cohort (reported in ref. [20]). Only transcripts with transcripts per million reads >1.0 were considered. Additionally, the database contained mutated peptide sequences including single amino acid alterations (SAAAs) from a curated list of somatic alterations identified by whole genome- or whole exome sequencing (WGS/WES) on the same cohort (reported in ref. [19]). Potential novel peptides were identified at class-specific false discovery rate (FDR) < 0.01. The novel peptides were further filtered by searching against a larger collection of reference protein databases followed by SpectrumAI [8]. To investigate if peptides from gene fusions could be found, a curated list of gene fusions detected from RNA-seq data of the same cohort was used.

Limma was used to identify differences in the levels of novel peptides between diagnosis and relapse samples [22]. A paired design matrix was applied, where each diagnosis sample was matched against its patient-matched relapse sample (adult: *n* = 22 diagnosis/relapse pairs; pediatric: *n* = 16 diagnosis/relapse pairs). Only peptides with more than one peptide spectrum match (PSM) were considered.

### Statistics

Throughout this study, significance was defined as FDR < 0.01 for peptide identification and *P* < 0.05 for two-sided tests unless otherwise stated.

## Results

**Fig. 1:**
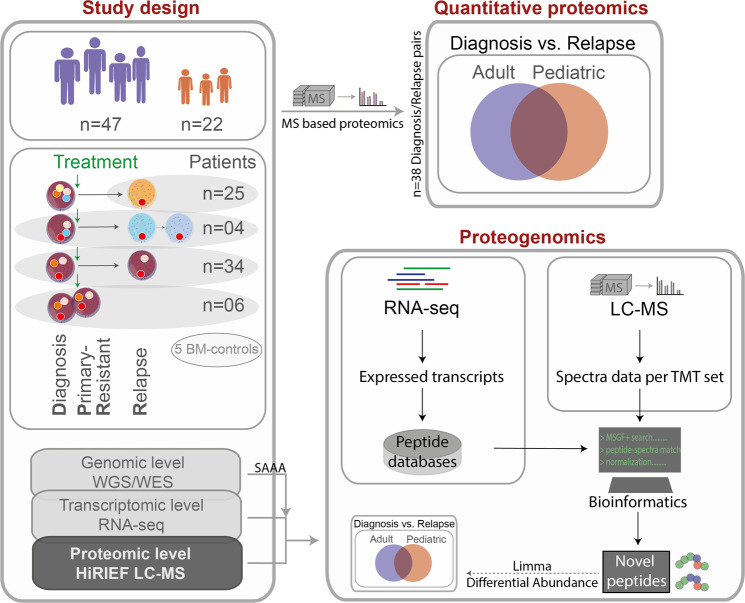
Proteomic and proteogenomic workflow. RNA-seq data were generated through ref. [20], and the WES/WGS data generated as part of ref. [19]. A detailed description of the steps included in the right-hand panel of this figure is given in Supplementary Fig. 8, and in the following sub-sections in the Supplementary Methods: “Sample processing and in-depth proteomics by using HiRIEF LC-MS”, “Peptide identification and quantification” and “Proteogenomic identification of novel peptides and single amino acid alterations”. *BM-controls* CD34-expressing bone marrow samples from healthy individuals, *LC-MS* liquid chromatography mass spectrometry, *RNA-seq* transcriptome sequencing, *SAAA* single amino acid alteration, *TMT* tandem mass tags, *WES* whole exome sequencing, *WGS* whole genome sequencing.

To further our knowledge of the R/PR AML proteome, we analyzed the peptide composition of 119 AML samples from 47 adult and 22 pediatric R/PR AML cases by HiRIEF LC-MS. The cohort comprised samples collected at diagnosis (*n* = 42) and relapse (*n* = 71), as well as PR samples (*n* = 6), including 38 patient-matched diagnosis-relapse pairs, and four diagnosis-PR pairs (Table 1 and Supplementary Tables 2 and 3). All of the herein included samples had a known genomic background based on WGS or WES [19], with RNA-seq-based transcriptomic data [20] available for all besides one of the samples (Supplementary Table 7). HiRIEF LC-MS- and RNA-seq data from five individual BM-controls were used as normal controls for proteomic and proteogenomic analyses. A schematic overview of the experimental workflow and downstream analyses is given in Fig. 1.

### Overview of the R/PR AML proteome

We quantified peptides matched to 10662 proteins, which were annotated to 10070 distinct genes in adult samples, and to 10218 proteins annotated to 9726 genes in pediatric samples, based on gene symbol centric quantification (denoted proteins henceforth). To discover recurrent proteome changes between diagnosis and relapse, a subset of proteins (adult: *n* = 6797; pediatric: *n* = 6926; Supplementary Table 6) annotated to 6717 genes that were quantified in each adult sample, and to 6840 genes in each pediatric sample, was used for quantitative proteome analyses. An intersection of 6094 annotated genes were identified between the adult and pediatric cohort.

For an overview of the proteomes, we applied unsupervised principal component analysis (PCA) on all AML- and BM-control samples part of the cohort (Supplementary Fig. 1). Adult patient AML008-associated samples showed a highly distinct proteome compared to all other adult samples (Supplementary Fig. 1A), expected to be caused by a partial or total loss of ten different chromosomes, resulting in hypodiploid AML. This case was therefore excluded from the downstream differential protein abundance analysis. As expected, the unsupervised PCA revealed that all BM-control samples formed a distinct group (Supplementary Fig. 1A), meanwhile sequential tumor samples from the same patient mainly clustered together (Supplementary Fig. 1B, C). No distinct clusters could be detected with regards to underlying mutational groups, such as *DNMT3A, NPM1* or internal tandem duplication in *FLT3* (data not shown), as also previously reported [14].

### Proteins involved in mitochondria-associated pathways are enriched at relapse

**Fig. 2:**
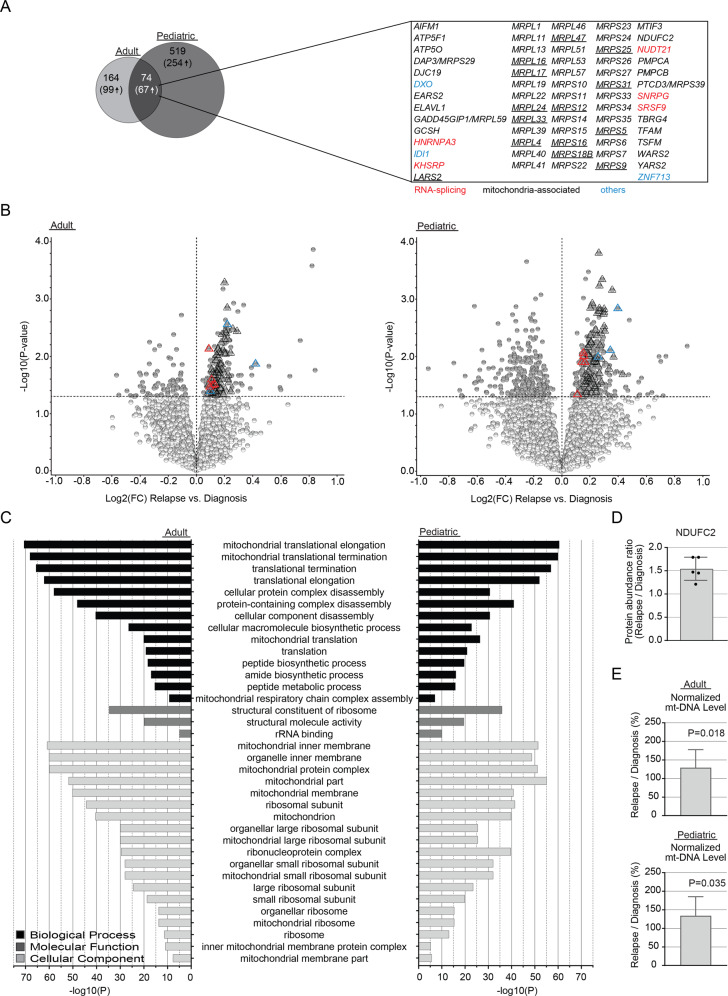
Proteins with altered abundance associated with AML relapse. **A** Left: Venn diagram depicting the intersection of diagnosis versus relapse-specific proteins with significantly altered abundance (*P* < 0.05) between the adult and pediatric cohorts. The number of significantly altered proteins are indicated, with numbers within parentheses specifying proteins upregulated at relapse. In the right-hand panel, the gene annotation for the intersection of significantly altered proteins upregulated at relapse in adults and children is listed. Proteins linked to mitochondrial functions are shown in black, those associated to RNA-splicing are highlighted in red, with the rest being depicted in blue. Underlined proteins indicate the proteins part of the intersection between the adult and pediatric cohort and the corresponding differential protein abundance analysis performed by Aasebø et al. [18]. **B** Volcano plots presenting proteins with altered abundance with proteins downregulated (log2FC < 0) and upregulated (log2FC > 0), respectively, at relapse in comparison to patient-matched diagnosis samples, for adult (left) and pediatric (right) cases. Proteins with altered abundance following *P* < 0.05 are highlighted in dark gray. Triangles indicate the intersection of significantly altered proteins upregulated at relapse in adults and children, as shown in panel **A**. **C** GO-analysis of relapse-associated significantly altered proteins for adult (left) and pediatric (right) cases. All presented GO-terms are enriched among proteins upregulated at relapse, compared to paired diagnosis samples (*P* < 0.05). Only shared GO-terms between the adult and pediatric cohort, with an FDR < 0.01 and a minimum enrichment score of three, are included. **D** Bar diagrams presenting the mean protein abundance ratio (relapse/diagnosis) based on densitometry analysis of immunoblots of NDUFC2, after normalization to the β-Actin loading control. Original immunoblots and case-based protein abundance ratios are presented in Supplementary Fig. 3. **E** Bar diagrams presenting the mean ratio of mitochondrial DNA read depth over the mean read depth of the nuclear genome, as presented by the ratio at relapse divided by the ratio at diagnosis. Visualization and underlying statistical calculations were performed by using Qlucore omics explorer v.3.6. (**A** and **B**), the Gene Ontology enRIchment anaLysis and visuaLizAtion tool (**C**), and GraphPad v.9.1.2 (**D** and **E**). For **E**, the applied statistical test was Non-parametric One sample Wilcoxon signed rank test with theoretical median = 100. Supplementary Table 8A–D presents details regarding samples included in this figure, Supplementary Table 9 presents details for all proteins with altered abundance and Supplementary Table 10 presents details for all GO-terms. *FC* fold change, *FDR* false discovery rate (Benjamini–Hochberg adjusted *P*-values), *GO* gene ontology, *mt* mitochondria.

To identify patterns within the AML proteome that define post-treatment AML, we compared altered protein abundances between patient-matched diagnosis and relapse samples (adult: *n* = 22 pairs; pediatric: *n* = 16 pairs; Supplementary Table 8A, B). This analysis resulted in 238 and 593 significantly altered proteins (*P* < 0.05) in adult and pediatric cases, respectively (Fig. 2A, Supplementary Table 9 and Supplementary Fig. 2), including 166 (adult) and 321 (pediatric) proteins with higher abundance at relapse. Seventy-four different proteins were found differentially expressed both in adults and children, with 67 of these being more abundant at relapse (Fig. 2A, B). Gene ontology enrichment analysis revealed overrepresentation of pathways involved in mitochondrial translation, peptide biosynthesis or mitochondrial respiratory chain complex assembly among proteins with higher abundance at relapse (Fig. 2C and Supplementary Table 10). Of the 67 proteins with higher abundance at relapse in both adults and children, 59 were mitochondria-associated. Among these, 23 and 19 were comprised of mitochondrial ribosomal proteins of the small, respectively large subunit (MRPS; MRPL; Fig. 2A). In addition, several proteins part of the mitochondrial respiratory chain complex were more abundant at relapse, including the NADH:Ubiquinone Oxidoreductase Core Subunit family (NDUF-A/B/C/S) (Supplementary Tables 9 and 10).

Further, in a comparison between our adult and pediatric datasets and a corresponding differential protein abundance analysis performed by Aasebø et al. [18] on seven AML diagnosis-relapse pairs, all of the 14 proteins part of the intersection between the three cohorts were constituted by mitochondria-associated proteins with higher abundance at relapse (Fig. 2A).

Finally, higher levels of mitochondria-associated proteins at relapse were verified by immunoblotting of MTIF3 and NDUFC2 for representative cases (Fig. 2D and Supplementary Fig. 3A–E).

Next, we investigated if higher abundance of mitochondria-associated proteins at relapse was linked to an increased number of mitochondria. Here, we utilized cohort-matched WGS-data, as the amount of mitochondrial DNA is expected to correlate with the amount of mitochondria per cell. This analysis revealed an approximately 30% higher abundance of mitochondrial DNA at relapse as compared to patient-matched diagnosis samples (Fig. 2E and Supplementary Figs. 3F, G and 4).

### Altered levels of RNA-splicing-related proteins during AML progression

**Fig. 3:**
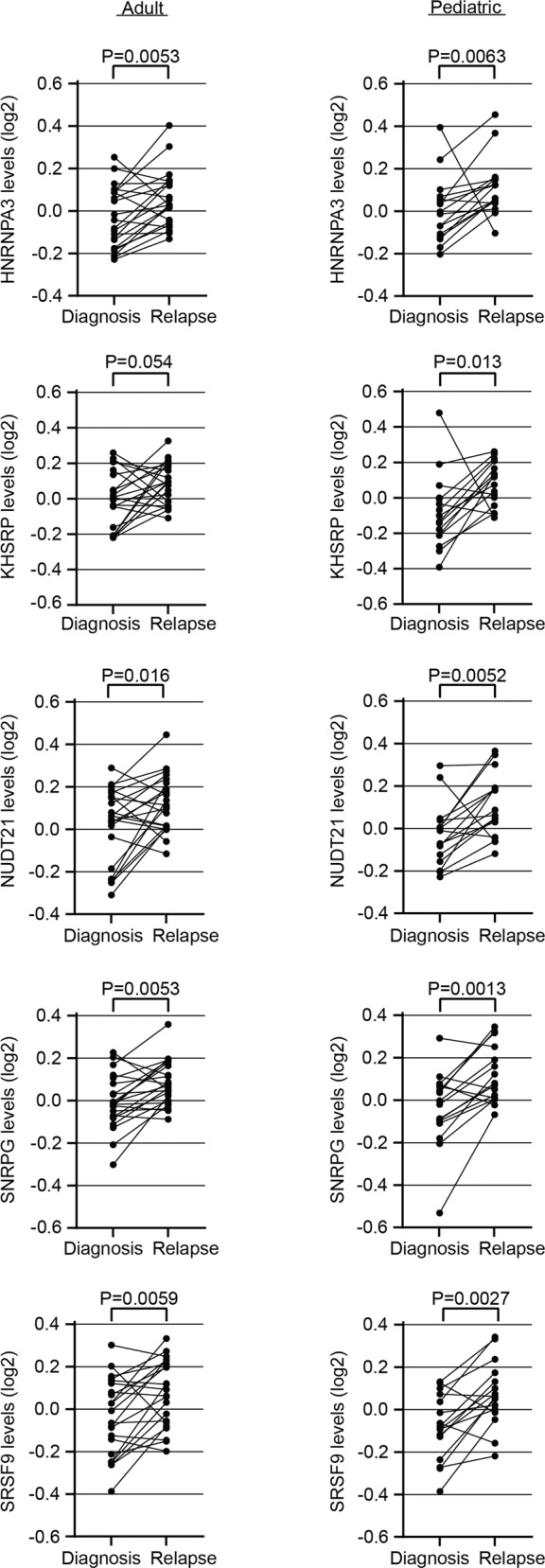
The levels of splicing-related proteins differ between AML diagnosis and relapse. Spaghetti plots presenting the protein levels (log2 transformed) in adult (left) and pediatric (right) AML, comparing paired diagnosis and relapse samples for the RNA-splicing related proteins HNRNPA3, KHSRP, NUDT21, SNRPG and SRSF9. Applied statistical test: Wilcoxon matched-pairs signed rank test. Visualization and underlying statistical calculations were performed by using GraphPad v.9.1.2. Supplementary Tables 8A, B presents details regarding samples included for generating the results presented in this figure.

Among the intersection of significantly altered proteins between adults and children, we also detected five RNA-splicing-related proteins (HNRNPA3, KHSRP, NUDT21, SNRPG and SRSF9; Fig. 2A). All of these proteins showed higher levels at relapse compared to diagnosis (Fig. 3 and Supplementary Fig. 5). Of note is that no correlation between the protein levels and sample-matched mRNA levels could be detected for any of the five proteins (Supplementary Table 11).

### Enrichment of a pro-inflammatory signature at AML relapse

**Fig. 4:**
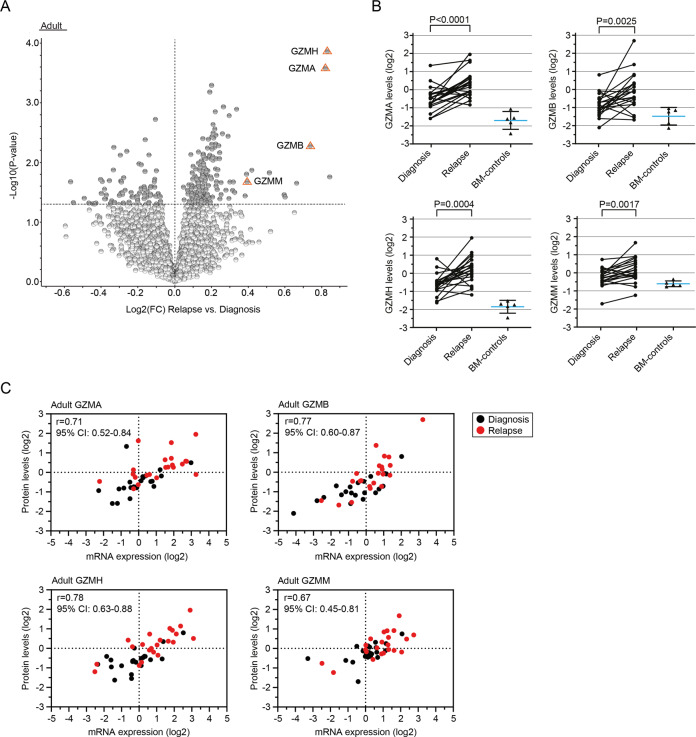
Upregulation of granzymes is linked to AML relapse. **A** Volcano plot presenting proteins with altered abundance with lower levels (log2FC < 0) and higher levels (log2FC > 0), respectively, at relapse in comparison to patient-matched diagnosis samples. Proteins with altered abundance following *P* < 0.05 are highlighted in dark gray, with orange triangles highlighting GZMA, GZMB, GZMH and GZMM among the highest ranked proteins. **B** Spaghetti plots presenting the protein levels (log2-transformed) in patient-matched diagnosis and relapse samples in adult AML for the granzymes GZMA, GZMB, GZMH and GZMM. Applied statistical test: Wilcoxon matched-pairs signed rank test. Each graph is overlaid with a scatter plot indicating mean and SD for BM-control samples of the respective granzyme. **C** Scatter plots depicting the correlation between log2-transformed protein levels and their sample-matched RNA expression levels (RNA-seq; TMM-normalized and log2-transformed) for the four different granzymes. Relapse samples are highlighted in red. Applied statistical test: Spearman correlation analysis. Visualization and underlying statistical calculations were performed by using Qlucore omics explorer v.3.6 (**A**) and GraphPad v.9.1.2 (**B** and **C**). Supplementary Table 8A presents details regarding samples included for generating the results presented in this figure, and Supplementary Table 11 presents details regarding statistical results associated with the correlation analysis between protein levels and mRNA-expression values. *BM-controls* CD34-expressing bone marrow samples from healthy individuals, *FC* fold change, *SD* Standard deviation, *TMM* Trimmed mean of M-values.

We detected four different granzymes (GZMA, GZMB, GZMH and GZMM) among the highest ranked proteins linked to relapse in adult AML patients (Fig. 4A, B and Supplementary Table 9). Elevated expression levels could be verified also at the transcriptomic level for all four granzymes, although not significant for *GZMB* (average Spearman *R* = 0.73; Fig. 4C, Supplementary Table 11 and Supplementary Fig. 6). Granzymes are serine-type peptidases and main regulators of cytotoxicity (reviewed in ref. [23]). More recently, however, they have also been linked to extracellular matrix remodeling [24] and inflammation [25, 26].

We previously identified lower mRNA expression of *CR1* (also called C3b/C4b-receptor or *CD35*) at relapse compared to patient-matched diagnosis samples both in adults (|log2FC| = 1.8; *P* = 0.0001) and children (|log2FC| = 1.6; *P* = 0.023; ref. [20]). Here, we could validate also significantly lower protein levels of CR1/CD35 at relapse compared to patient-matched diagnosis samples (adult: *P* = 0.0029; pediatric: *P* = 0.018; Supplementary Fig. 7), with a moderate to strong correlation between RNA- and protein levels (adult: Spearman *R* = 0.75, *P* < 0.0001; pediatric: Spearman *R* = 0.56, *P* = 0.001; Supplementary Table 11). CR1/CD35 is a negative regulator of the complement system. These findings further indicate towards the establishment of a pro-inflammatory signature, here also through complement activation [27, 28].

### Exploiting novel peptides as potential biomarkers or targets for immunotherapy in AML

Next, we applied a proteogenomic approach by taking advantage of the access to cohort-matched WGS/WES and RNA-seq data for investigation of potential novel and mutated peptides present in the proteomic data (Supplementary Fig. 8). To this end, we included the entire cohort to identify putative novel peptides found in diagnosis and/or R/PR samples. Cohort-matched RNA-seq data were analyzed at the peptide level by searching HiRIEF LC-MS spectra against a human canonical protein database appended with a customized peptide sequence database derived from transcriptomic data. A holistic approach was used, with the customized database containing all expressed transcripts detected from the cohort-matched RNA-seq data. Thus, this database contained theoretical peptides translated from non-canonical sequences covering, for instance, non-coding RNAs, pseudogenes, intron-retention, and exon extension, by performing three-reading-frame translation on assembled transcripts from the RNA-seq data.

**Fig. 5:**
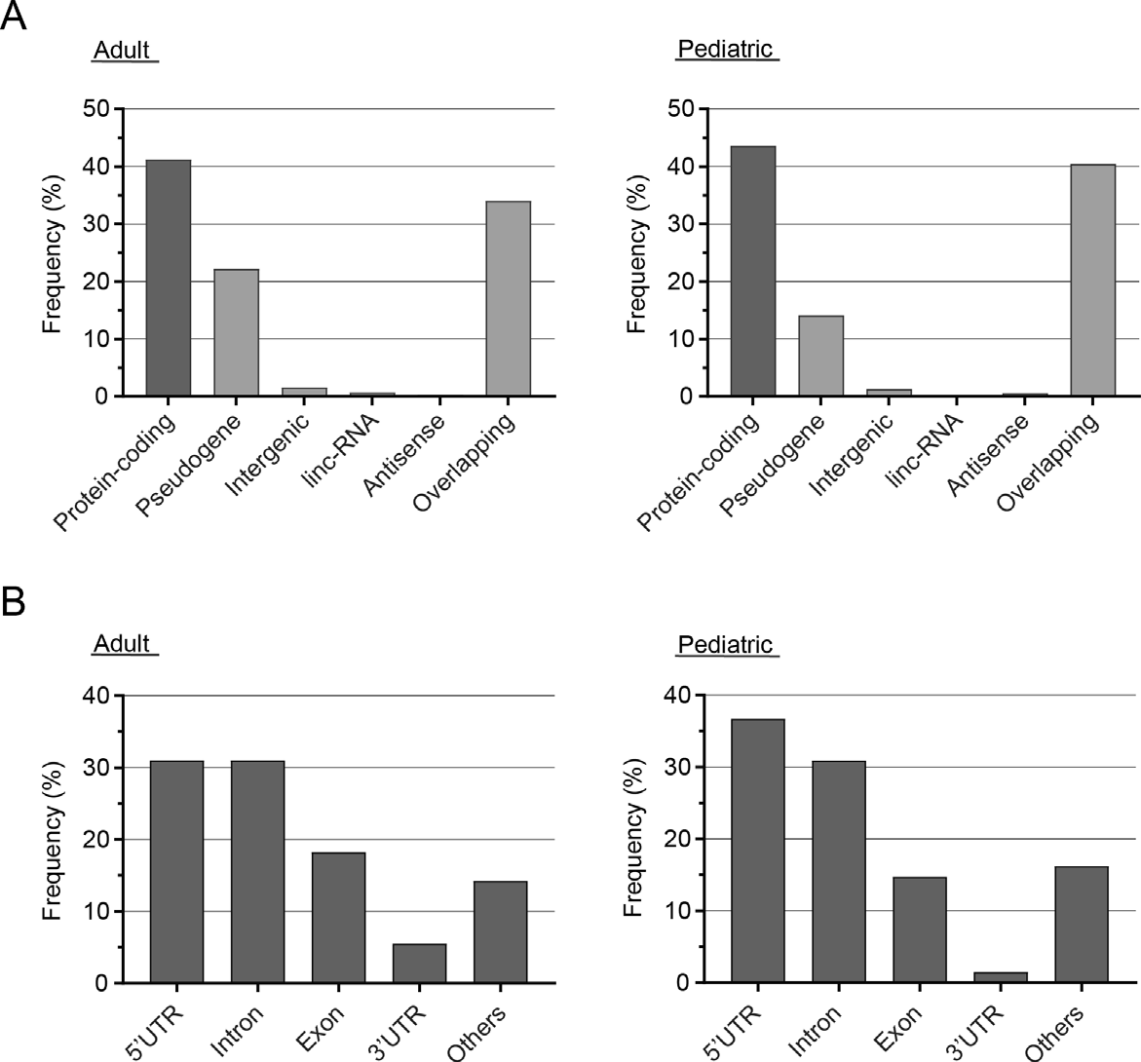
Frequencies of annotations for novel peptides derived from proteogenomic analyses. **A** Frequency of annotations for novel peptides detected in adult (left) and pediatric (right) R/PR AML samples via a proteogenomic approach. “Overlapping” indicates peptides annotated to multiple categories and/or genes. **B** Frequency of variants according to their location in relation to protein-coding genes. Only peptides annotated to one single protein-coding gene are included. “Others” refers to variants associated with non-canonical splicing or not otherwise specified. Supplementary Table 8E, F presents details regarding samples included for generating the results presented in this figure, and Supplementary Tables 12 and 13 present details for all novel peptides. *Linc-RNA* long intergenic noncoding RNAs, *UTR* untranslated region.

After blasting the data against human canonical protein sequences and removing peptides annotated to immunoglobulin (IG) genes, a total of 370 novel peptides (adult: *n* = 306; pediatric: *n* = 156) remained, with an intersection of 92 identical peptides found in both cohorts (Supplementary Tables 12 and 13). For the adult cohort, 126 novel peptides (41.2%) could confidently be annotated to 111 unique protein-coding genes. Further, 68 peptides were annotated to 55 pseudogenes (22.2%; Fig. 5A). The remaining peptides were either annotated to intergenic regions (*n* = 5), long non-coding RNAs (*n* = 2), antisense transcripts (*n* = 1), or to several potential transcripts simultaneously. For the pediatric cohort, 68 peptides (43.6%) were annotated to 55 unique protein-coding genes, with the remaining peptides being annotated to 22 pseudogenes (14.1%), two intergenic regions, one antisense transcript, and the rest to several potential transcripts simultaneously (Fig. 5A). Of the novel peptides that were confidently annotated to solely one protein-coding gene, 18.3% (adult), respectively 14.7% (children), were categorized as exon variants including, for instance, novel splice variants and extended exons. The remaining approximate 85% of the peptides were associated with untranslated regions (UTRs; adults: 5’-UTR *n* = 39 [31.0%], 3’-UTR *n* = 7 [5.6%]; children: 5’-UTR *n* = 25 [36.8%], 3’-UTR *n* = 1 [1.5%]), intronic regions (adults: *n* = 39 [31.0%]; children: *n* = 21 [30.9%]) or other genic regions (Fig. 5B).

By re-searching publicly available MS-data including seven AML diagnosis-relapse pairs [18], using a database supplemented with our 370 novel peptides, we could confirm the existence of 58 of the novel peptides (Supplementary Tables 12 and 13).

Thereafter, we sought to identify outlier samples per peptide sequence to identify sample-specific sequences. This analysis revealed 57 unique peptides in 45 different adult AML-samples (max: *n* = 10 peptides for AML042-R1) passing the outlier threshold (two times third-quantile; excluding IG-genes; Supplementary Fig. 9). For children, 20 unique peptides in 14 AML-samples passed the threshold (max: *n* = 3 for AML072-D and AML103-R1; Supplementary Fig. 10). No association was found between the number of outlier peptides per sample and the disease stage or specific mutations, including mutations in spliceosome-associated genes.

In addition, we identified a total of 25 novel peptides (adult: *n* = 16; pediatric: *n* = 9) with significantly different abundance between relapse- and patient-matched pre-treatment samples (*P* < 0.05; PSM > 1; Supplementary Tables 14 and 15). Among these, most peptides were annotated as translated pseudogenes or resulted from novel translational start sites.

### Detection of SAAAs and fusion peptides through a proteogenomic approach

As a means to investigate the potential of using HiRIEF LC-MS for detection of single nucleotide variants (SNV), we examined the overlap between alterations identified at the DNA- or RNA level and our proteomic data. For this, a similar approach as for detection of novel peptides was utilized. Tumor-derived sequences were analyzed at the peptide level by searching HiRIEF LC-MS spectra against a customized database of mutated peptides. To generate the customized database, six-frame translation of SNVs detected from the WGS/WES data (*n* = 1160 unique SNVs; ref. [19]) was applied.

Based on the customized database containing mutated peptides, 14 SAAAs (adult: *n* = 12; pediatric: *n* = 2) were detected by searching our MS-dataset (Supplementary Tables 7, 12 and 13). Of specific note is a p.R98H mutation in *GDI2* (GDP Dissociation Inhibitor 2) found in AML008-D and AML008-R1 by HiRIEF LC-MS, whereas only identified in AML008-D by WES analysis of these samples. Manual inspection of the WES reads by using Integrative Genomics Viewer [29] confirmed the presence of this missense variant in 32% of the reads. For confident detection of a larger fraction of translated SNVs by MS, however, an even higher sensitivity would be required.

Finally, we generated a database of fusion peptides by performing a three-frame translation of fusion transcripts detected from the RNA-seq data [20]. Searching the MS-data based on the customized database containing 57 fusion transcripts previously reported for cases with available sample-matched RNA-seq and HiRIEF LC-MS data, two different protein fusions could be validated in three patients (KMT2A::ELL in AML057; NSD1::NUP98 in AML071 and AML085; Supplementary Table 16).

## Discussion

Although recent genomic and transcriptomic studies have helped tremendously in elucidating the molecular alterations driving AML initiation, alterations on these levels often cannot be directly translated into molecular functions. The proteome, however, has the benefit of representing the combined consequences of genetic, transcriptomic and epigenetic events. To the best of our knowledge, sequential tumor samples from just seven adult AML patients have previously been analyzed at the proteomic level, without the incorporation of proteogenomic data [18]. Here, we report the first proteogenomic analysis of both adult and pediatric R/PR AML. The study is comprised of HiRIEF LC-MS, WGS/WES and RNA-seq data from 69 AML patients, aimed at identifying indications on how dysregulated and altered proteins mediate relapse and therapy resistance in AML.

To identify novel protein signatures associated with AML progression, we compared protein expression patterns between pre-treatment diagnosis-samples and their patient-matched relapse samples, followed by assessment at the RNA-level. Our results (Fig. 2) strengthen the previously suggested hypothesis that the AML-proteome is characterized by dysregulated energy metabolism and RNA processing [14, 30, 31]. Whereas these former studies solely were based on diagnosis samples, we could here show that increased levels of mitochondria-associated proteins were even more pronounced at relapse. We found that 88% of the intersection of significantly altered proteins with increased protein abundance at relapse in adult and pediatric AML were comprised of mitochondria-associated proteins, including the MRPL/S-family (Fig. 2A). Of note is that the mitochondria-associated signature only was detected at the proteome level, with no indication towards upregulation at the mRNA level based on RNA-seq analysis [20]; a finding supported also by others [14]. Our finding with higher levels of mitochondria-DNA at relapse (Fig. 2E and Supplementary Fig. 3F, G), based on cohort-matched WGS-data, strongly suggests that the higher mitochondria-protein levels are due to more mitochondria in relapse cells, and not the result of increased translation of mitochondria-associated mRNA. The origin of these mitochondria is, though, still unknown. Recent studies suggest that AML- and breast cancer cells may hijack mitochondria from normal stromal- or immune cells via nanotube-mediated transfer [32, 33]. This strategy could be the underlying explanation for our findings. An increased mitochondrial activity and/or quantity might aid the tumor cells to satisfy their high energy demand [34]. In addition, leukemia stem cells (LSCs), in contrast to normal hematopoietic stem cells, are highly dependent on mitochondrial activity to regulate reactive oxygen species production [35]. As a result, inhibition of these pathways could lead to selective targeting of otherwise frequently resistant LSCs [36]. Supporting this, inhibition of mitochondrial translation and respiration can sensitize human leukemia cells to chemotherapeutic and targeted treatment [14, 37–42], as exemplified by the U.S. Food and Drug Administration-approved drug tigecycline, which showed selective anti-leukemic efficacy on human primary AML cells in vivo, especially in combination with Daunorubicin or Cytarabine [43]. Further, Venetoclax, which is a recent addition to the therapeutic repertoire in AML, targets LSCs through two independent mechanisms: firstly, through BCL-2 inhibition, and secondly, via suppression of metabolism and oxidative phosphorylation [44–46]. Overall, this highlights the potential of targeting mitochondria-associated pathways at AML relapse.

In addition, overrepresentation at relapse of proteins related to peptide synthesis suggests that increased translation might be associated to an enhanced risk of chemotherapy resistance. This is supported by a study showing that anthracycline-resistant AML cells became treatment responsive after exposure to protein synthesis inhibitors [47].

Functional annotation of relapse-associated genes revealed enrichment of proteins related to RNA-processing and splicing, with higher levels of HNRNPA3, KHSRP, NUDT21, SNRPG and SRSF9 at relapse compared to their diagnosis counterparts both for adults and children (Fig. 3). Additionally, we previously reported mutations in spliceosome-related genes in 14.6% and 8.0%, respectively, of adult- and pediatric R/PR AML cases, including mutations in *SF3B1/3, SRSF1/2/6, U2AF1* and *ZRSR2* (ref. [19]). Unique splice variants caused by alternative splicing are known to alter, for instance, proliferation, apoptosis, drug resistance, and invasion in cancer [48], with aberrant splicing potentially giving rise to novel tumor specific proteins. The serine/arginine-rich protein family, which is involved in alternative splicing, also regulates apoptosis by splicing of apoptosis-related genes (reviewed in refs. [48–51]. Together, this puts forward spliceosome-related proteins as potential therapeutic targets in AML, as previously suggested for other cancer types [52, 53].

Intracellular granzymes, in synergy with perforin, are well acknowledged for their key role in cytolysis (reviewed in ref. [23]). However, recent studies indicate more individual functions for each granzyme, especially when acting independent of perforin. In that setting, granzymes are suggested to act as soluble mediators of inflammation and immune response, and thus potentially promoting a tumor beneficial microenvironment through cytokine signaling [26]. We previously identified an association between AML relapse and activation of pro-inflammatory pathways at the RNA level [20]. Here, we report higher protein and RNA levels of GZMA, GZMB, GZMH and GZMM at relapse in adult AML compared to paired diagnosis samples, and healthy BM-controls (Fig. 4). Although functional studies are necessary to determine the role of each granzyme, the significantly higher protein levels at relapse underline a tumor-promoting role of these enzymes in AML.

Genomic, transcriptomic, epigenomic and proteomic data independently contribute to a better understanding of the biological differences between pre-treatment AML cells and their counterparts at relapse. However, a final goal would be to fully integrate data from several omic-levels. To this end, we integrated RNA-seq data with HiRIEF LC-MS-derived proteomic data to investigate tumor specific peptides. Through this proteogenomic approach, we identified 370 novel peptides and protein products representing, for instance, novel splice variants and translated pseudogenes and other non-protein-coding genomic regions, with 58 of these validated through another, smaller, AML MS-dataset [18] (Fig. 5 and Supplementary Tables 12–15). Among these, neoantigens represent a highly interesting new class of tumor-specific targets, with a particular focus on immunotherapeutic approaches.

Although out of the scope of this study, further analyses are needed to validate leukemia specificity among the detected novel peptides. Here, we included five individual BM-controls. As a result, a subset of the reported novel peptides was identified at the transcriptomic and/or proteomic level also in the BM-controls, highlighting the importance of including normal controls into multi-omics study approaches.

Collectively, our proteogenomic study suggests that distinct protein profiles can be linked to relapse, and may be associated with therapy resistance in AML. The identified protein profiles, together with AML-specific neoantigens, represent the foundation for the exploration of novel drugs and for maximizing the benefits of current treatments; altogether with the aim of improving outcome of R/PR AML patients.

## Supplementary information

Supplemental Information

Dataset 1

## Data Availability

The mass spectrometry proteomics data have been deposited to the ProteomeXchange Consortium via the Proteomics Identification Database – EMBL-EBI [54] partner repository with the dataset identifier PXD031107.

## References

[1] Dohner H, Estey E, Grimwade D, Amadori S, Appelbaum FR, Buchner T (2017). Diagnosis and management of AML in adults: 2017 ELN recommendations from an international expert panel. Blood..

[2] Estey EH (2018). Acute myeloid leukemia: 2019 update on risk-stratification and management. Am J Hematol.

[3] Stein EM, DiNardo CD, Pollyea DA, Fathi AT, Roboz GJ, Altman JK (2017). Enasidenib in mutant IDH2 relapsed or refractory acute myeloid leukemia. Blood..

[4] Stone RM, Mandrekar SJ, Sanford BL, Laumann K, Geyer S, Bloomfield CD (2017). Midostaurin plus Chemotherapy for Acute Myeloid Leukemia with a FLT3 Mutation. N Engl J Med.

[5] DiNardo CD, Stein EM, de Botton S, Roboz GJ, Altman JK, Mims AS (2018). Durable remissions with ivosidenib in IDH1-mutated relapsed or refractory AML. N Engl J Med.

[6] Branca RM, Orre LM, Johansson HJ, Granholm V, Huss M, Perez-Bercoff A (2014). HiRIEF LC-MS enables deep proteome coverage and unbiased proteogenomics. Nat Methods.

[7] Johansson HJ, Socciarelli F, Vacanti NM, Haugen MH, Zhu Y, Siavelis I (2019). Breast cancer quantitative proteome and proteogenomic landscape. Nat Commun.

[8] Zhu Y, Orre LM, Johansson HJ, Huss M, Boekel J, Vesterlund M (2018). Discovery of coding regions in the human genome by integrated proteogenomics analysis workflow. Nat Commun.

[9] Tong J, Helmy M, Cavalli FM, Jin L, St-Germain J, Karisch R, et al. Integrated analysis of proteome, phosphotyrosine-proteome, tyrosine-kinome, and tyrosine-phosphatome in acute myeloid leukemia. Proteomics. 2017;17. 10.1002/pmic.201600361 PMC5500908 28176486

[10] Foss EJ, Radulovic D, Stirewalt DL, Radich J, Sala-Torra O, Pogosova-Agadjanyan EL (2012). Proteomic classification of acute leukemias by alignment-based quantitation of LC-MS/MS data sets. J Proteome Res.

[11] Aasebø E, Berven FS, Bartaula-Brevik S, Stokowy T, Hovland R, Vaudel M, et al. Proteome and phosphoproteome changes associated with prognosis in acute myeloid leukemia. Cancers. 2020;12:709. 10.3390/cancers12030709 PMC7140113 32192169

[12] Nguyen NHK, Wu H, Tan H, Peng J, Rubnitz JE, Cao X, et al. Global proteomic profiling of pediatric AML: a pilot study. Cancers. 2021;13:3161. 10.3390/cancers13133161 PMC8268478 34202615

[13] Dowling P, Tierney C, Dunphy K, Miettinen JJ, Heckman CA, Bazou D (2021). Identification of protein biomarker signatures for acute myeloid leukemia (AML) using both nontargeted and targeted approaches. Proteomes..

[14] Jayavelu AK, Wolf S, Buettner F, Alexe G, Häupl B, Comoglio F (2022). The proteogenomic subtypes of acute myeloid leukemia. Cancer Cell.

[15] Casado P, Wilkes EH, Miraki-Moud F, Hadi MM, Rio-Machin A, Rajeeve V (2018). Proteomic and genomic integration identifies kinase and differentiation determinants of kinase inhibitor sensitivity in leukemia cells. Leukemia..

[16] Gosline SJC, Tognon C, Nestor M, Joshi S, Modak R, Damnernsawad A (2022). Proteomic and phosphoproteomic measurements enhance ability to predict ex vivo drug response in AML. Clin Proteom.

[17] Kramer MH, Zhang Q, Sprung R, Day RB, Erdmann-Gilmore P, Li Y (2022). Proteomic and phosphoproteomic landscapes of acute myeloid leukemia. Blood..

[18] Aasebø E, Berven FS, Hovland R, Døskeland SO, Bruserud Ø, Selheim F, et al. The progression of acute myeloid leukemia from first diagnosis to chemoresistant relapse: a comparison of proteomic and phosphoproteomic profiles. Cancers. 2020;12:1466. 10.3390/cancers12061466 PMC7352627 32512867

[19] Stratmann S, Yones SA, Mayrhofer M, Norgren N, Skaftason A, Sun J (2021). Genomic characterization of relapsed acute myeloid leukemia reveals novel putative therapeutic targets. Blood Adv.

[20] Stratmann S, Yones SA, Garbulowski M, Sun J, Skaftason A, Mayrhofer M (2022). Transcriptomic analysis reveals proinflammatory signatures associated with acute myeloid leukemia progression. Blood Adv.

[21] Moggridge S, Sorensen PH, Morin GB, Hughes CS (2018). Extending the compatibility of the SP3 paramagnetic bead processing approach for proteomics. J Proteome Res.

[22] Ritchie ME, Phipson B, Wu D, Hu Y, Law CW, Shi W (2015). limma powers differential expression analyses for RNA-sequencing and microarray studies. Nucleic Acids Res.

[23] Grossman WJ, Revell PA, Lu ZH, Johnson H, Bredemeyer AJ, Ley TJ (2003). The orphan granzymes of humans and mice. Curr Opin Immunol.

[24] Prakash Monica D, Munoz Marcia A, Jain R, Tong Philip L, Koskinen A, Regner M (2014). Granzyme B promotes cytotoxic lymphocyte transmigration via basement membrane remodeling. Immunity..

[25] Anthony DA, Andrews DM, Chow M, Watt SV, House C, Akira S (2010). A role for granzyme M in TLR4-driven inflammation and endotoxicosis. J Immunol.

[26] Metkar SS, Menaa C, Pardo J, Wang B, Wallich R, Freudenberg M (2008). Human and mouse granzyme A induce a proinflammatory cytokine response. Immunity..

[27] Hanahan D, Weinberg RA (2011). Hallmarks of cancer: the next generation. Cell..

[28] Iida K, Nussenzweig V (1983). Functional properties of membrane-associated complement receptor CR1. J Immunol.

[29] Robinson JT, Thorvaldsdóttir H, Winckler W, Guttman M, Lander ES, Getz G (2011). Integrative genomics viewer. Nat Biotechnol.

[30] Raffel S, Klimmeck D, Falcone M, Demir A, Pouya A, Zeisberger P (2020). Quantitative proteomics reveals specific metabolic features of acute myeloid leukemia stem cells. Blood..

[31] Aasebø E, Forthun RB, Berven F, Selheim F, Hernandez-Valladares M (2016). Global cell proteome profiling, phospho-signaling and quantitative proteomics for identification of new biomarkers in acute myeloid leukemia patients. Curr Pharm Biotechnol.

[32] Saha T, Dash C, Jayabalan R, Khiste S, Kulkarni A, Kurmi K, et al. Intercellular nanotubes mediate mitochondrial trafficking between cancer and immune cells. Nature Nanotechnology. 2022;17:98–106. 10.1038/s41565-021-01000-4 PMC10071558 34795441

[33] Saito K, Zhang Q, Yang H, Yamatani K, Ai T, Ruvolo V (2021). Exogenous mitochondrial transfer and endogenous mitochondrial fission facilitate AML resistance to OxPhos inhibition. Blood Adv.

[34] Mondet J, Lo Presti C, Chevalier S, Bertrand A, Tondeur S, Blanchet S, et al. Mitochondria in human acute myeloid leukemia cell lines have ultrastructural alterations linked to deregulation of their respiratory profiles. Exp Hematol. 2021;98:53–62.e3. 10.1016/j.exphem.2021.03.001 33689800

[35] Mattes K, Vellenga E, Schepers H (2019). Differential redox-regulation and mitochondrial dynamics in normal and leukemic hematopoietic stem cells: A potential window for leukemia therapy. Crit Rev Oncol/Hematol.

[36] Zhao LN, Björklund M, Caldez MJ, Zheng J, Kaldis P (2021). Therapeutic targeting of the mitochondrial one-carbon pathway: perspectives, pitfalls, and potential. Oncogene..

[37] Gross A, Katz SG (2017). Non-apoptotic functions of BCL-2 family proteins. Cell Death Differ.

[38] Sharon D, Cathelin S, Mirali S, Di Trani JM, Yanofsky DJ, Keon KA, et al. Inhibition of mitochondrial translation overcomes venetoclax resistance in AML through activation of the integrated stress response. Science translational medicine. 2019;11:eaax2863. 10.1126/scitranslmed.aax2863 31666400

[39] Farge T, Saland E, de Toni F, Aroua N, Hosseini M, Perry R (2017). Chemotherapy-resistant human acute myeloid leukemia cells are not enriched for leukemic stem cells but require oxidative metabolism. Cancer Disco.

[40] Salunkhe S, Mishra SV, Ghorai A, Hole A, Chandrani P, Dutt A (2020). Metabolic rewiring in drug resistant cells exhibit higher OXPHOS and fatty acids as preferred major source to cellular energetics. Biochim Biophys Acta Bioenerg.

[41] Carter JL, Hege K, Kalpage HA, Edwards H, Hüttemann M, Taub JW (2020). Targeting mitochondrial respiration for the treatment of acute myeloid leukemia. Biochemical Pharmacol.

[42] Stuani L, Sabatier M, Saland E, Cognet G, Poupin N, Bosc C, et al. Mitochondrial metabolism supports resistance to IDH mutant inhibitors in acute myeloid leukemia. J Exp Med. 2021;218:e20200924. 10.1084/jem.20200924 PMC7995203 33760042

[43] Skrtić M, Sriskanthadevan S, Jhas B, Gebbia M, Wang X, Wang Z (2011). Inhibition of mitochondrial translation as a therapeutic strategy for human acute myeloid leukemia. Cancer Cell.

[44] Lagadinou Eleni D, Sach A, Callahan K, Rossi Randall M, Neering Sarah J, Minhajuddin M (2013). BCL-2 inhibition targets oxidative phosphorylation and selectively eradicates quiescent human leukemia stem cells. Cell Stem Cell.

[45] Pollyea DA, Stevens BM, Jones CL, Winters A, Pei S, Minhajuddin M (2018). Venetoclax with azacitidine disrupts energy metabolism and targets leukemia stem cells in patients with acute myeloid leukemia. Nat Med.

[46] Roca-Portoles A, Rodriguez-Blanco G, Sumpton D, Cloix C, Mullin M, Mackay GM (2020). Venetoclax causes metabolic reprogramming independent of BCL-2 inhibition. Cell Death Dis.

[47] Gausdal G, Gjertsen BT, McCormack E, Van Damme P, Hovland R, Krakstad C (2008). Abolition of stress-induced protein synthesis sensitizes leukemia cells to anthracycline-induced death. Blood..

[48] Urbanski LM, Leclair N, Anczuków O (2018). Alternative-splicing defects in cancer: Splicing regulators and their downstream targets, guiding the way to novel cancer therapeutics. Wiley interdisciplinary reviews. RNA..

[49] Lin JC, Tsao MF, Lin YJ. Differential impacts of alternative splicing networks on apoptosis. Int J Mol Sci. 2016;17:2097. 10.3390/ijms17122097 PMC5187897 27983653

[50] Schwerk C, Schulze-Osthoff K (2005). Regulation of apoptosis by alternative pre-mRNA splicing. Mol Cell.

[51] de Necochea-Campion R, Shouse GP, Zhou Q, Mirshahidi S, Chen C-S (2016). Aberrant splicing and drug resistance in AML. J Hematol Oncol.

[52] Yoshino H, Enokida H, Chiyomaru T, Tatarano S, Hidaka H, Yamasaki T (2012). Tumor suppressive microRNA-1 mediated novel apoptosis pathways through direct inhibition of splicing factor serine/arginine-rich 9 (SRSF9/SRp30c) in bladder cancer. Biochemical Biophysical Res Commun.

[53] Zhang Q, Lv R, Guo W, Li X (2019). microRNA-802 inhibits cell proliferation and induces apoptosis in human cervical cancer by targeting serine/arginine-rich splicing factor 9. J Cell Biochem.

[54] Perez-Riverol Y, Csordas A, Bai J, Bernal-Llinares M, Hewapathirana S, Kundu DJ (2018). The PRIDE database and related tools and resources in 2019: improving support for quantification data. Nucleic Acids Res.

