# Supplementary material for: Proteogenomic analysis of acute myeloid leukemia associates relapsed disease with reprogrammed energy metabolism both in adults and children
Source: Leukemia. 2022 Dec 26;37(3):550–9. doi: 10.1038/s41375-022-01796-7 (PMC9991901; doi:10.1038/s41375-022-01796-7)
Supplement: Supplementary file 1 — Supplemental Information [file 41375_2022_1796_MOESM1_ESM.pdf]

SUPPLEMENTAL INFORMATION FOR

Proteogenomic analysis of acute myeloid leukemia associates relapsed disease with reprogrammed energy metabolism both in adults and children

Svea Stratmann[1], Mattias Vesterlund[2], Husen M. Umer[2], Saeed Eshtad[1,3], Aron Skaftason[4], Morten Krogh Herlin[5,6], Christer Sundström[1], Anna Eriksson[7], Martin Höglund[7], Josefine Palle[8], Jonas Abrahamsson[9], Kirsi Jahnukainen[10], Monica Cheng Munthe-Kaas[11,12], Bernward Zeller[12], Katja Pokrovskaja Tamm[13], Cecilia Lindskog[1], Lucia Cavelier[1], Janne Lehtiö[2], Linda Holmfeldt[1,14,*]

[1]Department of Immunology, Genetics and Pathology, Science for Life Laboratory, Uppsala University, Sweden.

[2]Department of Oncology-Pathology, Karolinska Institutet, and Science for Life Laboratory, Stockholm, Sweden.

[3]Center for Hematology and Regenerative Medicine, Department of Medicine, Karolinska Institutet, Stockholm, Sweden.

[4]Department of Molecular Medicine and Surgery, Karolinska Institutet, Stockholm, Sweden

[5]Department of Clinical Medicine, Aarhus University, Denmark.

[6]Department of Pediatrics and Adolescent Medicine, Aarhus University, Denmark.

[7]Department of Medical Sciences, Uppsala University, Sweden.

[8]Department of Women's and Children's Health, Uppsala University, Sweden.

[9]Department of Pediatrics, Institute of Clinical Sciences, Sahlgrenska Academy at University of Gothenburg, Gothenburg, Sweden.

[10]Children's Hospital, University of Helsinki and Helsinki University Central Hospital, Helsinki, Finland.

[11]Norwegian Institute of Public Health, Oslo, Norway.

[12]Division of Pediatric and Adolescent Medicine, Oslo University Hospital, Oslo, Norway.

[13]Department of Oncology and Pathology, Karolinska Institutet and Karolinska University hospital, Sweden.

[13]The Beijer Laboratory, Uppsala, Sweden.

[*]Corresponding author

TABLE OF CONTENTS

SUPPLEMENTAL METHODS 4
- Cohort information 4
- Sample processing and in-depth proteomics by using HiRIEF LC-MS 5
- Peptide identification and quantification 6
- Tumor purity assessment 7
- Protein abundance analysis, and calculation and visualization of statistical significance 7
- Gene ontology enrichment analysis 8
- Immunoblotting 8
- Assessment of mitochondrial DNA levels 9
- Proteogenomic identification of novel peptides and single amino acid alterations 9
- Detection of fusion peptides 10
- Identification of samples expressing the respective novel peptide 10
- Manual curation of identified novel peptides 11
- Code availability 11
- Sample usage for various analyses 11

SUPPLEMENTAL TABLE LEGENDS 12
- Supplemental Table 1: Clinical information 12
- Supplemental Table 2: Study cohort sample characteristics 12
- Supplemental Table 3. Study cohort samples and applied analysis methods. 12
- Supplemental Table 4: BM-control sample characteristics. 13
- Supplemental Table 5: Antibody information. 13
- Supplemental Table 6. Expressed proteins and annotated genes in adult and pediatric R/PR AML. 13
- Supplemental Table 7. Comprised metadata and multi-omics results overlay. 13
- Supplemental Table 8. Sample usage for various analyses. 13
- Supplemental Table 9. Proteins with altered abundance between diagnosis and relapse samples. 14
- Supplemental Table 10. GO-analysis of relapse-associated significantly altered proteins. 14
- Supplemental Table 11. Spearman correlation analysis between protein and mRNA levels. 14
- Supplemental Table 12. Novel peptides derived from proteogenomic analysis of the adult cohort. 14
- Supplemental Table 13. Novel peptides derived from proteogenomic analysis of the pediatric cohort. 14
- Supplemental Table 14. Novel peptides with altered abundance between adult diagnosis and paired relapse samples. 15
- Supplemental Table 15. Novel peptides with altered abundance between pediatric diagnosis and paired relapse samples. 15
- Supplemental Table 16. Fusion peptides derived from proteogenomic analysis. 15
- Supplemental Table 17. HiRIEF fractions gradient length. 15

Supplemental Table 18. WGS-based read depth of mitochondrial and nuclear DNA. 15

SUPPLEMENTAL FIGURES 16

Supplemental Figure 1: Unsupervised clustering of adult and pediatric R/PR AML. 16

Supplemental Figure 2: Proteins with significantly altered levels between paired diagnosis and relapse samples 18

Supplemental Figure 3: Higher levels of mitochondria-associted proteins and mitochondrial DNA at AML relapse 19

Supplemental Figure 4: Higher levels of mitochondrial DNA at AML relapse. 21

Supplemental Figure 5: The levels of splicing-related proteins differ between AML diagnosis and relapse 22

Supplemental Figure 6: Upregulation of granzymes at AML relapse is detected also at the transcriptomic level 23

Supplemental Figure 7: Lower protein and mRNA expression of CR1 at AML relapse. 24

Supplemental Figure 8. Overview of the proteogenomic workflow 25

Supplemental Figure 9: Highly abundant novel peptides in adult R/PR AML. 27

Supplemental Figure 10: Highly abundant novel peptides in pediatric R/PR AML. 29

SUPPLEMENTAL REFERENCES 30

SUPPLEMENTAL METHODS

Cohort information

Acute myeloid leukemia (AML) samples (n=119) were retrieved from Uppsala Biobank or the Karolinska Institute Biobank, with all of the samples being collected from 1995 through 2016. The following sample collections part of Uppsala Biobank were utilized: i) Clinical Pathology, Uppsala University Hospital, Sweden; ii) Nordic Society of Paediatric Haematology and Oncology (www.nopho.org); and iii) U-CAN(1). Further, a sample collection at Astrid Lindgren's Children's Hospital, Stockholm, Sweden, that is part of the Karolinska Institute Biobank, was utilized. All patients included in this study were clinically characterized and classified following common standards (refs.(2, 3) and NOPHO-DBH AML 2012 Protocol [EudraCT Number 2012-002934-35]).

Event-free survival (EFS) was determined as the time from initial diagnosis until first relapse or initial treatment failure, with the latter indicated by time = 0. Short EFS was defined as < 6 months for adults and < 12 months for pediatric patients. Primary resistant (PR) cases suffered treatment failure without reaching first complete remission. Persistent relapse (R-P) samples were acquired post-relapse treatment from patients not achieving complete remission after relapse onset.

Genomic characterization of the entire study cohort, in the form of whole genome sequencing (WGS) or whole exome sequencing (WES), was reported previously(4) (data available via controlled access: doi.org/10.17044/scilifelab.12292778), including bone marrow (BM) derived normal stromal cells as well as complete remission BM samples as a source of germline DNA. The analyzed WGS/WES data comprise information about somatic single nucleotide variants (SNVs), insertions and deletions (InDels), copy number alterations and copy-neutral loss-of-heterozygosity(4). Further, transcriptomic characterization in the form of transcriptome sequencing (RNA-seq) was previously reported(5) for all besides one of the samples, including CD34-expressing BM cells from five healthy donors serving as normal controls (BM-controls; **Supplemental Table 4**; data available via controlled access: doi.org/10.17044/scilifelab.13105229). The analyzed RNA-seq data comprise differential gene expression results and identified gene fusions, SNVs and small InDels (as part of ref.(5)).

Detailed clinical and biological characteristics are summarized in **Supplemental Tables 1-3**.

Sample processing and in-depth proteomics by using HiRIEF LC-MS

Proteomic analysis was performed by high-resolution isoelectric focusing liquid chromatography mass spectrometry (HiRIEF LC-MS) on 119 AML samples and five BM-control samples (**Supplemental Tables 3** and **4**). The sample source composition was as follows: BM: n=71/119, 59.7%; Peripheral blood (PB): n=26/119, 21.8%; Unknown if BM or PB: n=19/119, 16.0%; BM and PB sample pooled after processing due to insufficient amount of lysate if kept separated: n=2/119, 1.7%; Pleural fluid: n=1/119, 0.84% (**Supplemental Table 2**).

Precipitated protein fractions from the AllPrep DNA/RNA/Protein kit (Qiagen, Hilden, Germany) were dissolved in 50µl Lysis buffer (4% SDS, 20mM HEPES pH 7.6, 1mM DTT), heated to 95°C and sonicated. The total protein amount was estimated by using the DC Protein Assay (Bio-Rad, Hercules, CA, USA). Samples were prepared for MS analysis by using a modified version of the SP3 protein clean-up and digestion protocol(6, 7), where proteins were digested by Lys-C and trypsin (sequencing grade modified, Pierce™ Thermo Fisher Scientific, Waltham, MA, USA). In brief, 71-250µg protein from each sample was reduced by addition of 1mM DTT and alkylated with 2.5mM Iodoacetamide. Sera-Mag SP3 bead mix (10µl) was added together with acetonitrile (final concentration 50%). The mix was incubated under rotation at room temperature for eight minutes and placed on a magnetic rack. The supernatant was discarded, followed by two washes with 70% ethanol and one with 100% acetonitrile. The beads-protein mixture was reconstituted in 50µl Lys-C buffer (1M Urea, 25mM HEPES pH 7.6) and incubated overnight. Finally, trypsin was added in 1:50 enzyme to protein ratio in 50µl 25mM HEPES pH 7.6 and incubated overnight. The peptides were eluted from the mixture after placing the mixture on a magnetic rack, followed by peptide concentration measurement (DC Protein Assay).

The samples were thereafter pH adjusted by using TEAB pH 8.5 (100mM final concentration). Forty µg of peptides from each sample were labelled with isobaric tandem mass tags (TMT10plex reagent; Thermo Fisher Scientific) according to the manufacturer's protocol and thereafter separated by immobilized pH gradient - isoelectric focusing (IPG-IEF) on 3–10 strips as described previously(8). Samples were pooled into sets of ten as detailed in **Supplemental Table 2**. Each set comprised of adult samples included a BM-control sample or a technical control together with up to eight different tumor samples and a pooled sample (internal standard; IS). To be able to compare and/or integrate the data derived from the adult and the pediatric samples, the pooled IS sample of the adult MS-analysis (referred to as IS-Adult) was

included also in pediatric set B. Additionally, all other sets comprised of pediatric samples included a technical control. The labelling efficiency was determined by LC-MS/MS before pooling of the samples. For the sample clean-up step, a solid phase extraction (SPE strata-X-C, Phenomenex, Torrance, CA, USA) was performed and purified samples were dried in a SpeedVac. An aliquot of approximately 10µg was suspended in LC mobile phase A and 1µg was injected on the LC-MS/MS system.

Online LC-MS was performed as previously described(8) by using a Dionex UltiMate™ 3000 RSLCnano System coupled to a Q-Exactive-HF mass spectrometer (Thermo Fisher Scientific). All samples were dissolved in 20µl solvent A and subsequently 10µl were injected. Samples were trapped on a C18 guard-desalting column (Acclaim PepMap 100, 75µm x 2 cm, nanoViper, C18, 5µm, 100Å), and separated on a 50cm long C18 column (Easy spray PepMap RSLC, C18, 2µm, 100Å, 75µm x 50cm). The nano capillary solvent A was 94.9% water, 5% DMSO, 0.1% formic acid; and solvent B was 4.9% water, 5% DMSO, 90% acetonitrile, 0.1% formic acid. At a constant flow of 0.25µl per minute, the curved gradient went from 6-8% B up to 40% B in each fraction in a dynamic range of gradient length (**Supplemental Table 17**), followed by a steep increase to 100% B in 5 min. FTMS master scans with 60000 resolution (and mass range 300-1500m/z) were followed by data-dependent MS/MS (30000 resolution) on the top five ions by using higher energy collision dissociation at 30% normalized collision energy. Precursors were isolated with a 2m/z window. Automatic gain control targets were 1e6 for MS1 and 1e5 for MS2. Maximum injection times were 100ms for MS1 and 100ms for MS2. The entire duty cycle lasted ~2.5s. Dynamic exclusion was used with 30s duration. Precursors with unassigned charge state or charge state one were excluded. An underfill ratio of 1% was used.

Peptide identification and quantification

Orbitrap raw MS/MS files were converted to mzML format by using msConvert from the ProteoWizard tool suite(9). Spectra were subsequently searched by using MS-GF+ (release 2017.07.21; ref.(10)) and Percolator (v.3.01.01; ref.(11)), with search results from eight subsequent fractions being grouped for Percolator target/decoy analysis. For the adult cohort, searches were done against the human protein subset of Ensembl v.75 in a Galaxy(12) proteomics workflow (v.2.3; ref.(13)). For the pediatric cohort, searches were done against the human protein subset of Ensembl v.75 in a Nextflow (v.19.04.0) proteomics workflow. Of importance is that Galaxy and Nextflow only serve as workflow executors for the softwares

utilized in the searches, and the same softwares were contained in both pipelines. In both workflows, MS-GF+ settings included precursor mass tolerance of 10ppm, fully-tryptic peptides, maximum peptide length of 50 amino acids and a maximum charge of six. Fixed modifications were TMT10plex on lysines and peptide N-termini, and carbamidomethylation on cysteine residues. A variable modification setting was used for oxidation on methionine residues. Quantification of TMT10plex reporter ions was done by using OpenMS(14) project's IsobaricAnalyzer. Peptide spectrum matches (PSMs) found at 1% false discovery rate (FDR) were used to infer gene identities.

Protein quantification by TMT10plex reporter ions was calculated by using PSM TMT ratios to the internal standard and normalized to the sample median. The median PSM TMT reporter ratio from peptides unique to a gene symbol was used for quantification. Protein FDRs were calculated by utilizing the picked-FDR(15) method by using gene symbols as protein groups and limited to 1% FDR.

Tumor purity assessment

Next-generation sequencing-based tumor purity assessment was manually performed based on patient-matched genomic material(4) extracted together with the corresponding proteins (**Supplemental Table 2**). For samples sequenced by WGS, purity was based on their available somatic genomic aberrations, and how the relative effect on sequence coverage (deletion from two to one copy), the allele ratio of heterozygous single nucleotide polymorphisms (in regions with copy-neutral loss-of-heterozygosity) and somatic SNVs (in diploid regions) would theoretically scale with tumor purity. The estimated purity for samples sequenced by WES was based on solely somatic SNVs present in diploid regions. The next-generation sequencing-based purity results were further compared to information from morphology-based purity assessment of May Grünwald and Giemsa stained cells post cryopreservation and, if applicable, post immune-based depletion of non-tumor cells (**Supplemental Table 2**).

Protein abundance analysis, and calculation and visualization of statistical significance

Qlucore omics explorer 3.6 was utilized to perform data correction for sex, for generating an overview of the proteomic landscape, and for analysis of differential protein levels between diagnosis and relapse samples. The following visualizations and statistical calculations were performed by using Qlucore omics explorer v.3.6 with default settings, if not otherwise specified: Principal Component Analysis (PCA; according to ref.(16-18)); t-Distributed Stochastic Neighbor Embedding (t-SNE; according to ref.(19, 20)); Hierarchical clustering and

associated heat maps were constructed following ref.(21), by using log2-transformed normalized values following the Euclidean metric on normalized variables (mean = 0, variance = 1). Further, genes were ranked according to their R/R^2-statistic values (R-statistics; **Supplemental Table 9**), which Qlucore computes according to the coefficient of partial determination. The Benjamini-Hochberg(22) method was applied to correct for multiple testing and the fold change (FC) was calculated from the difference between the arithmetic averages over each group. Volcano plots were used to identify the highest ranked proteins with altered abundance among sample groups and Venn diagrams were utilized to inspect the intersection of significantly altered proteins between experiments.

GraphPad Prism v.9.1.2 and v.9.2.0 were used to calculate significant differences observed in protein, mRNA and DNA levels between two or more groups, and results were visualized in the form of scatter plots with mean and standard deviation (SD), spaghetti plots or bar diagrams. The Mann-Whitney test was used for two-group comparisons on non-parametric data, whereas the Kruskal-Wallis test was used for multi-group comparisons on non-parametric data followed by Dunn´s correction for multi-group comparisons. For a patient-matched two-group comparison on non-parametric data, the Wilcoxon matched-pairs signed rank test was applied and results were shown in the form of spaghetti plots. For one group comparisons, the non-parametric one sample Wilcoxon signed rank test with theoretical median = 1.0 or 100, as appropriate, was used. Correlation between protein levels and mRNA expression values of matched samples was calculated by using the non-parametric measure of Spearman's rank correlation coefficient (**Supplemental Table 11**).

Gene ontology enrichment analysis

Gene Ontology (GO) enrichment analysis was carried out by using Gene Ontology enRIchment anaLysis and visuaLizAtion tool (GOrilla(23, 24)). A target list of upregulated proteins was compared to the background of all detected proteins (adult: n = 6797; pediatric: n = 6926; **Supplemental Table 6**) by using the standard Hyper Geometric statistics with a P-value threshold of 0.01. Significant GO-terms with Benjamini-Hochberg adjusted P-values (FDR < 0.01) and a minimum enrichment score of three, were displayed in the form of bar diagrams (**Supplemental Table 10**).

Immunoblotting

Total cell lysates were denatured in NuPAGE™ LDS Sample Buffer (NP0007; Thermo Fisher Scientific) at 70°C for 10 min. At least 36µg of total lysate per sample were separated via SDS-

PAGE (NuPAGE™ 4 to 12%, Bis-Tris gels; NP0323BOX; Thermo Fisher Scientific). Proteins were transferred to nitrocellulose membranes using iBlot™ transfer stacks (IB301002; Thermo Fisher), followed by blocking with Phosphate Buffered Saline containing 0.05% (v/v) Tween-20 and 5% skimmed milk for 1h at room temperature, and overnight incubation at 4°C with primary antibodies (anti- β-Actin [1:5000 dilution; A5441; Sigma-Aldrich; Target protein size: 42kDa], anti-MTIF3 [1:500; HPA039791; Atlas Antibodies; Target protein size: 32kDa], anti-NDUFC2 [1:500 dilution; 15573-1-AP; Proteintech; Target protein size: 14kDa]; **Supplemental Table 5**). Incubation with secondary antibodies (Amersham ECL anti-mouse IgG-HRP conjugate [1:5000 dilution; NA931; Cytiva], Amersham ECL anti-rabbit IgG-HRP conjugate [1:5000 dilution; NA934; Cytiva]) was carried out at room temperature for 45min. Pierce™ ECL Western Blotting Substrate (32106; Thermo Fisher) was used for horseradish peroxidase (HRP)-conjugated secondary antibodies. Images were acquired and analyzed using an Amersham™ Imager 680 (GE Healthcare). Densitometry analysis of protein levels was performed using the Amersham™ Imager 680 associated software, and the values were normalized to the respective β-Actin loading control.

Assessment of mitochondrial DNA levels

For investigation of the relative frequency of mitochondrial DNA (mtDNA) per AML sample, all cases with available patient-matched diagnosis- and relapse WGS data(4) (**Supplemental Table 8C-D**) were examined with regards to read depth of mtDNA and nuclear DNA, the latter as assessed via the mean read depth of the entire nuclear genome, as well as of the two mitochondria-associated genes *MTIF3* and *NDUFC2*, which are located in the nuclear genome. The mitochondrial and genomic read depth for the respective samples are presented in **Supplemental Table 18**. By dividing [mtDNA read depth] with [mean nuclear genome read depth], the sample-specific and normalized ratio of the amount of mtDNA versus nuclear DNA was achieved for each sample. Thereafter, a case-specific ratio was generated by dividing the [relapse normalized ratio] with the [diagnosis normalized ratio] for each case, rendering information on alterations in the mtDNA amount over time for each case.

Proteogenomic identification of novel peptides and single amino acid alterations

Transcripts were assembled from the RNA-seq data(5) of each sample by using StringTie (v.2.1; ref.(25)) based on the human reference gene annotations (Ensembl v.75). Next, transcripts with low expression level (transcript per million < 1) were removed and the remaining transcripts were three-frame translated into protein sequences by using the

Biopython (v1.72; ref.(26)) package. Additionally, a curated list of somatic mutations identified from patient-matched WGS/WES derived data(4) was used to generate mutated peptide sequences (SAAAs). Each mutation was extended by 50bp at both sides and the obtained DNA sequence from GRCh37 was used as the reference DNA. The corresponding positions in the reference sequence were altered to obtain a mutated sequence. Next, each of the reference and the altered sequences were six-frame translated.

The protein sequences from the transcripts and mutations were combined and digested into tryptic peptides. Tryptic peptides with a minimum length of eight amino acids and a maximum length of 40 amino acids were retained. The database was fractionated based on the peptide isoelectric points as further detailed by Branca *et al.*(8). Finally, the human canonical proteins (Ensembl v.75) were appended to the peptide database.

The proteomics data from each cohort were searched against the peptide database from the same cohort by using MS-GF+ (v.2020.01.15; ref.(10)). Percolator (v.3.04.0 ref.(11)) was used for Percolator target-decoy scoring. Peptides at FDR < 0.01 were considered significant, while those matching canonical protein sequences were removed. By using blast, the remaining peptides were searched against a larger collection of reference protein databases that included Uniprot (as of December 11, 2019); ref.(27), Gencode v.33 ref.(28), Ensembl v.99 ref.(29), and RefSeq (as of May 29, 2020). Peptides matching sequences from the collection of reference protein databases were removed and those with one mismatch were further validated by using SpectrumAI(30). Finally, the list of novel peptides contained peptides with more than one mismatch or no match to known proteins, as well as those peptides that passed SpectrumAI validation (**Supplemental Tables 12** and **13**).

Detection of fusion peptides

A list of curated gene fusions identified by RNA-seq were translated into protein sequences by three-frame translation. For FDR calculation purposes, the protein sequences were reversed to obtain a decoy set of sequences. The two protein sets (fusion-translations and their decoys) were appended to the human canonical protein sequences (Ensembl v.75). Fusion peptides that did not match any canonical proteins were considered as novel peptides.

Identification of samples expressing the respective novel peptide

To identify the sample(s) in which the respective novel peptide was the most abundant, the TMT ratio for all channels across the TMT sets for each novel peptide sequence per cohort was

obtained. In order to normalize the values across the sets, the mean ratio was calculated by dividing the sum of all channels by the number of channels excluding the reference channel as well as channels with *NA* values. Next, a threshold was defined as the third quantile multiplied by two in order to detect samples with high expression levels. Finally, only samples that had a normalized expression larger than the defined threshold were considered to have the respective peptide highly expressed. Results were visualized in the form of box plots showing the normalized expression level per peptide sequence across all samples where the peptide was detected (**Supplemental Figures 8** and **9**).

Manual curation of identified novel peptides

The output of the proteogenomic search was further manually curated. Here, variants annotated as "exon_variant", "five_prime_utr_extension", "intron_retention" or "other" with a simultaneous annotation as "canonical" with regards to reading frame, were manually investigate by using the UCSC genome browser(31). Through this process, ten variants identified in adults and three variants identified in children were excluded, since they were identical to an already reported unverified isoform (n = 11), or to the expected resulting peptide based on a known common single nucleotide polymorphism (n = 2).

Code availability

Custom codes are available from the authors upon request.

Sample usage for various analyses

Detailed information regarding samples included in the comparison between diagnosis and paired relapse samples, as well as those part of the proteogenomic analyses, is present in **Supplemental Table 8**.

SUPPLEMENTAL TABLE LEGENDS

Supplemental Tables 1-18 are present in a separate Supplemental document, including a content list, abbreviations and a legend regarding color coding on the first sheet of that document.

Supplemental Table 1: Clinical information. Clinical characteristics of the patients included in the R/PR AML study cohort are summarized. Allo, allogeneic; Auto, autologous; CR, complete remission; D, diagnosis; F, female; HSCT, hematopoietic stem cell transplantation; M, male; M0-M7, the French-American-British (FAB) classification of AML; MDS, myelodysplastic syndromes; NOS, not otherwise specified; PR, primary resistant; R1/2/3, sequential relapses; t-AML, treatment related AML; WBC, white blood cell count; VP, current treatment protocol.

Supplemental Table 2: Study cohort sample characteristics. Detailed information regarding the characteristics of the 119 tumor samples and five BM-control samples included in the current study. Sample purity and cell viability are given in intervals of 10 and 25%, respectively. Included are also the HiRIEF LC-MS set composition and information about technical controls. Allo, allogeneic; BM, bone marrow; BM-controls, CD34-expressing BM cells from healthy donors; D, diagnosis; HSCT, hematopoietic stem cell transplantation; HiRIEF LC-MS, high resolution isoelectric focusing liquid chromatography mass spectrometry; NOPHO, Nordic Society of Paediatric Haematology and Oncology; PB, peripheral blood; PR, primary resistant; R1/2/3, sequential relapses; R1/2-P, persistent relapse specimen; RNA-seq, transcriptome sequencing; U-CAN, Uppsala Umeå Comprehensive Cancer Consortium, Sweden; WES, whole exome sequencing; WGS, whole genome sequencing.

Supplemental Table 3. Study cohort samples and applied analysis methods. Summary of the AML samples included in the study cohort, as well as overlapping genomic and transcriptomic analysis methods performed for the respective samples. BMS, bone marrow derived stromal cells; CR, complete remission; D, diagnosis; G, genomic (WGS/WES) data available; PR, primary resistant; R1/2/3, sequential relapses; R1/2-P, persistent relapse specimen; P, proteomic data available; T, transcriptomic (RNA-seq) data available; WES, whole exome sequencing; WGS-30X, whole genome sequencing, aiming at > 30X coverage; WGS-90X, whole genome sequencing, aiming at > 90X coverage.

Supplemental Table 4: BM-control sample characteristics. Summary of the characteristics of the five individual healthy donors and associated samples (CD34-expressing bone marrow cells; ABM017F; AllCells Inc) included in the study cohort.

Supplemental Table 5: Antibody information. Antibodies used for purification of patient-derived AML samples by immune-based depletion of non-tumor cells, as well as for immunoblotting.

Supplemental Table 6. Expressed proteins and annotated genes in adult and pediatric R/PR AML. Quantified proteins detected in all adult and/or pediatric AML samples alongside with their corresponding gene annotation are listed. The list of proteins was utilized for differential protein expression analyses and as background list for the gene ontology and enrichment investigations.

Supplemental Table 7. Comprised metadata and multi-omics results overlay. Various sample- and clinical information, combined with data regarding variants recurrently identified in the R/PR cohort, on a per sample basis. Next-generation sequencing data are based on WGS, WES and RNA-seq results reported previously(4, 5). Information in regards to sample usage for various analyses is included for each sample. CNA, copy number alteration; CN-LOH, copy-neutral loss-of-heterozygosity; D, diagnosis; F, female; FAB, the French-American-British classification of AML; InDel, insertion or deletion mutation; M, male; MS, mass spectrometry; PR, primary resistant; R1/2/3, sequential relapses; R-P, persistent relapse specimen; RNA-seq, transcriptome sequencing; WES, whole exome sequencing; WGS-30X, whole genome sequencing, aiming at > 30X coverage; WGS-90X, whole genome sequencing, aiming at > 90X coverage.

Supplemental Table 8. Sample usage for various analyses. Tumor samples included for various analyses. **A**-**B**) Protein abundance analyses between samples associated with relapse versus paired pre-treatment diagnosis, of (A) adult and (B) pediatric R/PR AML samples. **C-D**) Samples used for the assessment of sample-specific mitochondrial DNA abundance based on patient-matched diagnosis and relapse samples with available WGS-data for adults (C) and children (D). **E-F**) Samples used for the proteogenomic analyses, separated into (E) adult and (F) pediatric samples. BM-controls, CD34-expressing bone marrow cells from healthy donors; D, diagnosis; PR, primary resistant; R1/2/3, sequential relapses; R-P, persistent relapse specimen; WGS, whole genome sequencing.

Supplemental Table 9. Proteins with altered abundance between diagnosis and relapse samples. Summarized are all proteins with altered abundance and the corresponding annotated genes between diagnosis and relapse samples with a P-value < 0.05. FC > 1.0 indicates higher protein levels at relapse compared to diagnosis, meanwhile FC < 1.0 indicates lower protein levels. Only patient-matched diagnosis and relapse samples were included in the comparison (adult: n = 22 diagnosis-relapse pairs; pediatric: n = 16 diagnosis-relapse pairs). **Supplemental Table 8A-B** presents details regarding samples included for generating the results presented in this table. FC, fold change; FDR, false discovery rate; R-statistic, square roots of the R2-statistics (coefficient of determination).

Supplemental Table 10. GO-analysis of relapse-associated significantly altered proteins. Detailed results of the GO enrichment analysis, by utilizing GOrilla, for relapse versus paired diagnosis samples. Proteins with altered abundance with a P-value < 0.05 and a FC > 1 were analyzed against the background of all expressed proteins found in all adult, respectively, pediatric samples (**Supplemental Table 6**). B, total number of genes associated with a specific GO term; b, number of genes in the intersection; FC, fold change; FDR, false discovery rate (Benjamini–Hochberg adjusted P-values); GO, gene ontology; N, total number of protein-coding genes (background list); n, number of genes in the target set.

Supplemental Table 11. Spearman correlation analysis between protein and mRNA levels. Correlation analysis-associated statistics between protein levels and mRNA-expression values for GZMA, GZMB, GZMH, GZMM, CR1, HNRNPA3, KHSRP, NUDT21, SNRPG, and SRSF9.

Supplemental Table 12. Novel peptides derived from proteogenomic analysis of the adult cohort. Listed are all novel peptides derived from the proteogenomic analysis after filtration against UniProt, Ensembl, GENCODE and RefSeq. **Supplemental Table 8E** presents details regarding AML samples included for generating the results presented in this table. “Also identified in data from Aasebø *et al.*“ refers to ref.(32). D, diagnosis; PR, primary resistant; PSM, peptide spectrum match; R1/2/3, sequential relapses.

Supplemental Table 13. Novel peptides derived from proteogenomic analysis of the pediatric cohort. Listed are all novel peptides derived from the proteogenomic analysis after filtration against UniProt, Ensembl, GENCODE and RefSeq. **Supplemental Table 8F** presents details regarding AML samples included for generating the results presented in this table. “Also

identified in data from Aasebø *et al.*“ refers to ref.(32). D, diagnosis; PR, primary resistant; PSM, peptide spectrum match; R1/2/3, sequential relapses; R1/2-P, persistent relapse specimen.

Supplemental Table 14. Novel peptides with altered abundance between adult diagnosis and paired relapse samples. Listed are all novel peptides derived from the proteogenomic analysis on adult AML, with different abundance levels between paired diagnosis and relapse samples, based on limma. A log fold change (logFC) above zero indicates increased peptide abundance at relapse relative to diagnosis. **Supplemental Table 8A** presents details regarding AML samples included for generating the results presented in this table. “Also identified in data from Aasebø *et al.*“ refers to ref.(32). IG, immunoglobulin; PSM, peptide spectrum match; SAAA, single amino acid alteration.

Supplemental Table 15. Novel peptides with altered abundance between pediatric diagnosis and paired relapse samples. Listed are all novel peptides derived from the proteogenomic analysis on pediatric AML, with different abundance levels between paired diagnosis and relapse samples, based on limma. A log fold change (logFC) above zero indicates increased peptide abundance at relapse relative to diagnosis. **Supplemental Table 8B** presents details regarding AML samples included for generating the results presented in this table. “Also identified in data from Aasebø *et al.*“ refers to ref.(32). IG, immunoglobulin; PSM, peptide spectrum match; SAAA, single amino acid alteration.

Supplemental Table 16. Fusion peptides derived from proteogenomic analysis. Listed are all fusion peptides derived from the proteogenomic analysis by using customized databases generated from RNA-seq-derived fusion transcripts. D, diagnosis; PR, primary resistant; PSM, peptide spectrum match; R1/2/3, sequential relapses; R1/2-P, persistent relapse specimen.

Supplemental Table 17. HiRIEF fractions gradient length. Given are detailed information about sample fractions and corresponding gradient length.

Supplemental Table 18. WGS-based read depth of mitochondrial and nuclear DNA. Given is the read depth of mitochondrial and nuclear DNA based on previously published WGS data(4) for the indicated samples. Nuclear DNA read depth is given both as a mean of the entire nuclear genome, as well as for the two mitochondria-associated genes *MTIF3* and *NDUFC2,* which are located in the nuclear genome. D, diagnosis; R1/2/3, sequential relapses; R1/2-P, persistent relapse specimen; WGS, whole genome sequencing.

SUPPLEMENTAL FIGURES

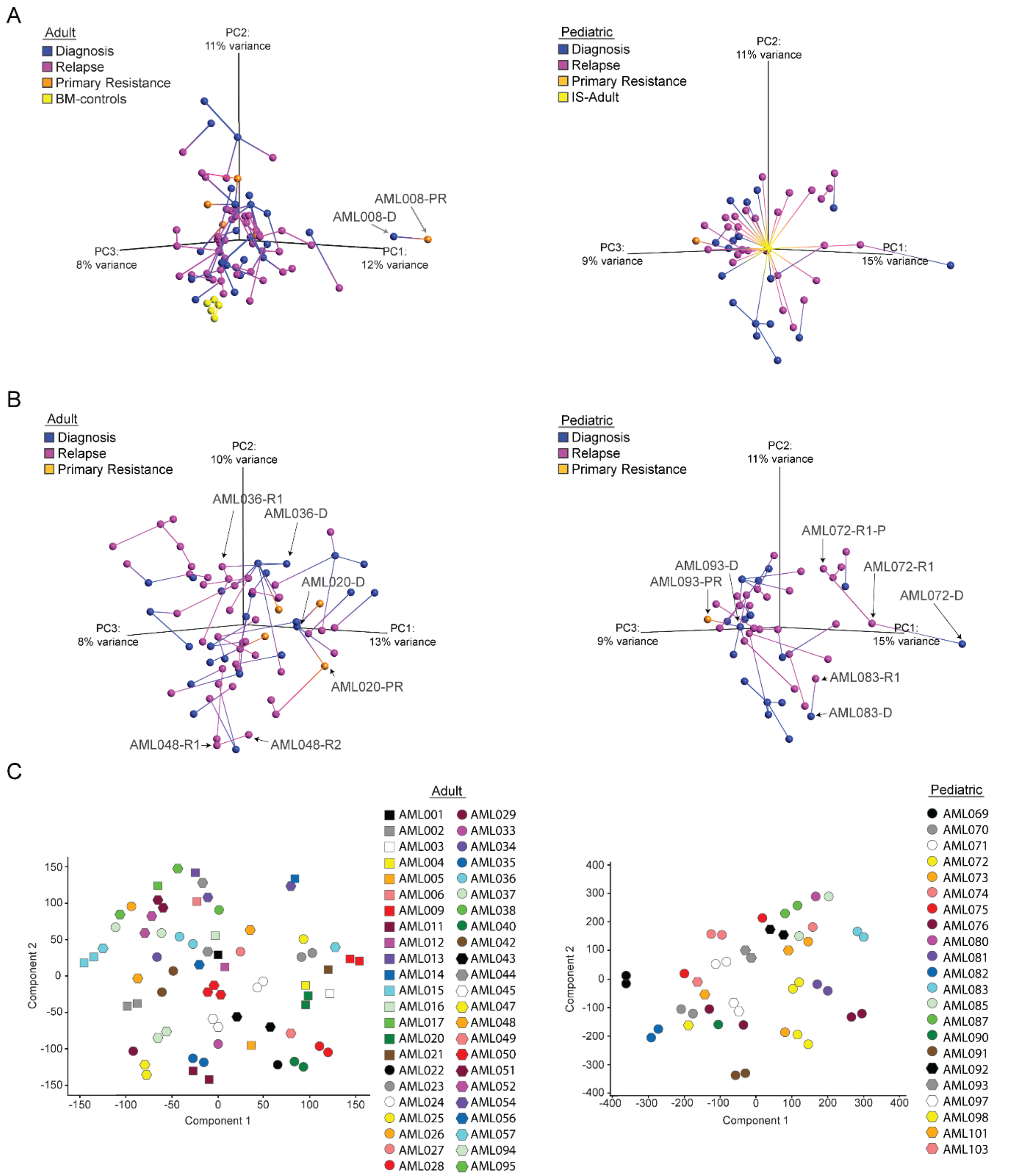

Supplemental Figure 1: Unsupervised clustering of adult and pediatric R/PR AML. A-B) Principal component analysis (PCA) of tumor and BM-control proteomics data. **A**) PCA with all samples part of the (left) adult R/PR AML cohort including the BM-control samples, and (right) the pediatric cohort including the internal standard (IS-Adult). **B**) PCA plot for (left) adult AML samples represented by 6797 proteins, post exclusion of the outlier AML008-D/PR and the BM-control samples, as well as (right) pediatric samples post removal of the internal

standard pool (IS-Adult), represented by 6926 proteins. **C**) Neighboring information in the form of T-distributed stochastic neighbor embedding (t-SNE) plots showing similarity between sequential patient-matched tumor samples in adults (left; perplexity = 7) and children (right; perplexity = 5). Visualization and underlying calculations were performed by using Qlucore omics explorer v.3.6. BM-controls, CD34-expressing bone marrow cells from healthy donors; IS-Adult, internal standard formed by a pool of all adult AML samples that all pediatric samples were normalized against, and thus used as an internal control.

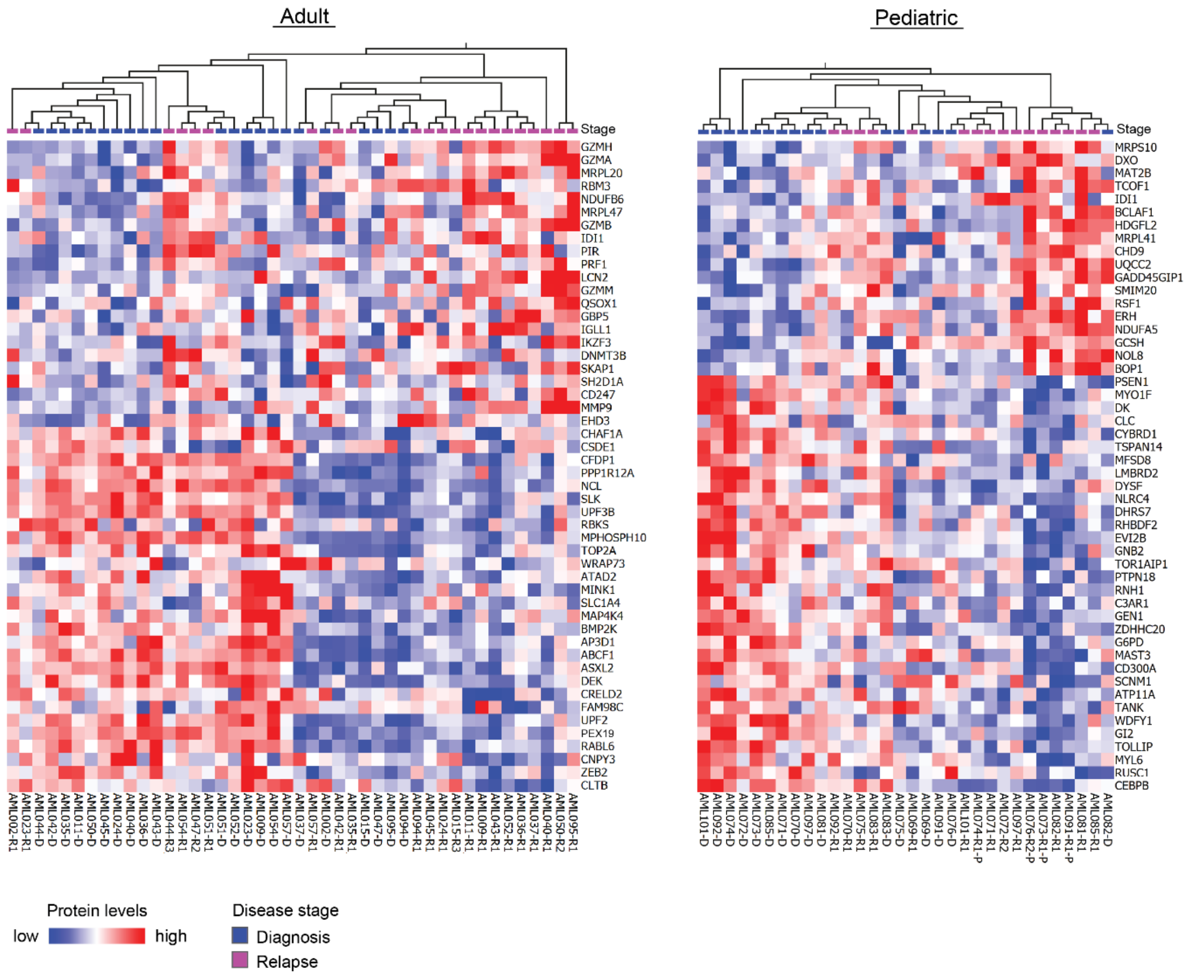

Supplemental Figure 2: Proteins with significantly altered levels between paired diagnosis and relapse samples. Heat maps and hierarchical cluster analysis of the top 50 ranked significantly altered proteins between paired diagnosis and relapse samples for adult (left) and pediatric (right) cases (X-axis). Proteins are ranked according to their R-statistic values (Y-axis), with higher protein levels at relapse depicted in red and lower protein levels in blue. Gene annotation information is given at the right side of each heat map. Visualization and underlying statistical calculations were performed by using Qlucore omics explorer v.3.6. **Supplemental Table 8A-B** presents details regarding samples included for generating the results presented in this figure, and **Supplemental Table 9** presents a detailed list including all significantly altered proteins ($P < 0.05$). D, diagnosis; R1/2/3, relapse 1/2/3; R1/2-P, persistent relapse.

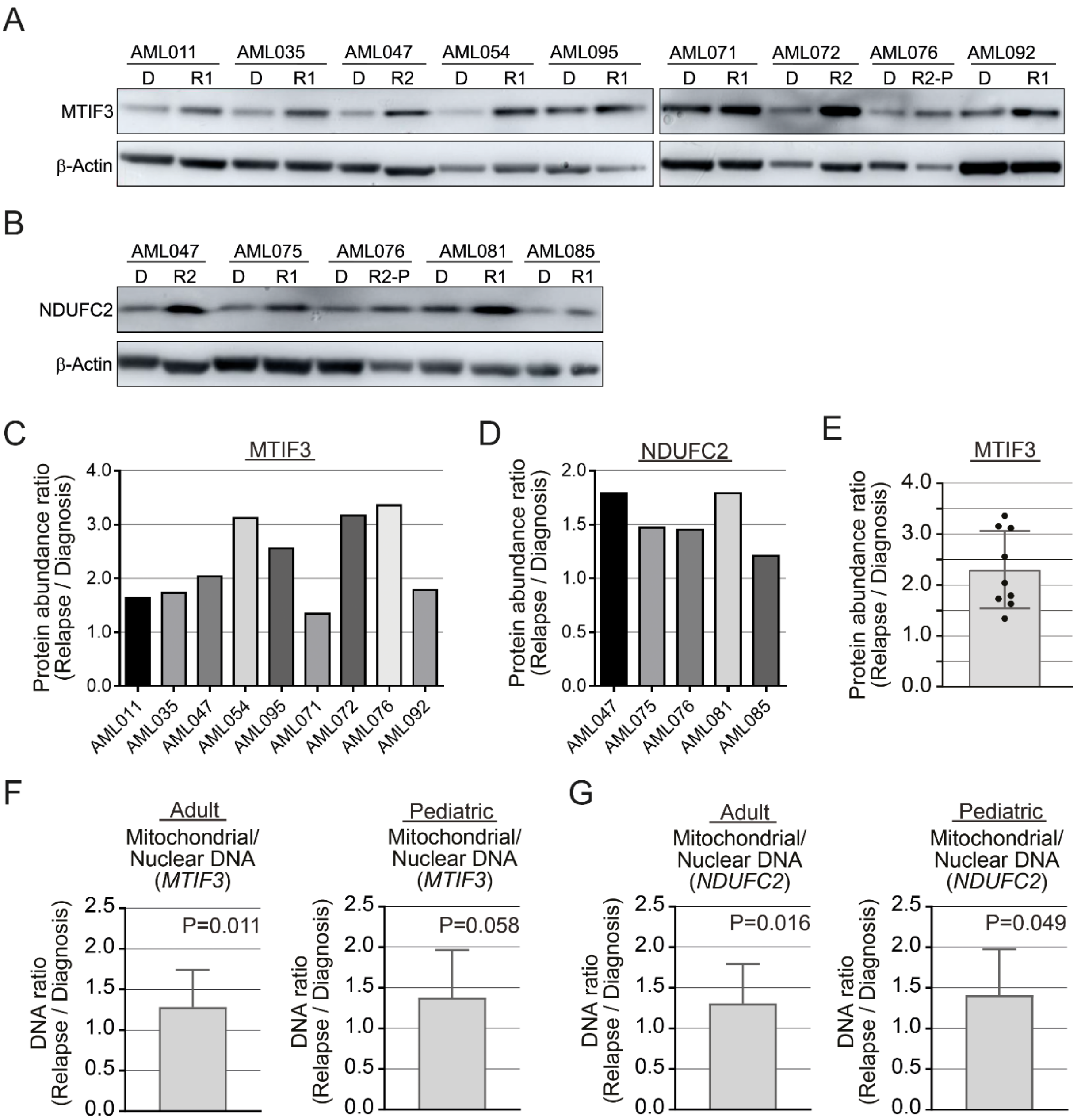

Supplemental Figure 3: Higher levels of mitochondria-associted proteins and mitochondrial DNA at AML relapse. A-B) Immunoblot analysis of the mitochondria-associated proteins MTIF3 (A; antibody HPA039791; Atlas Antibodies) and NDUFC2 (B; antibody 15573-1-AP; Proteintech) on total cell lysates (≥36µg) from patient-matched diagnosis and relapse samples from representative AML cases as indicated in the figure. β-Actin (antibody A5441; Sigma-Aldrich) was used as loading control. **C-D**) Bar diagrams showing the MTIF3 (C) and NDUFC2 (D) protein abundance ratio (relapse / diagnosis) based on densitometry analysis of the immunoblots in (A) and (B), respectively, followed by normalization to the respective β-Actin loading control. **E**) Bar diagram presenting the mean

protein abundance ratio (relapse / diagnosis) based on densitometry analysis of immunoblots of MTIF3, after normalization to the β-Actin loading control. Original immunoblots and case-based protein abundance ratios are presented in (A). **F-G**) Bar diagrams (Left, Adults; Right, Children) presenting the mean ratio of the mitochondrial DNA read depth over the read depth of the mitochondria-associated genes *MTIF3* (F) and *NDUFC2* (G), which are located in the nuclear genome, as presented by the ratio at relapse divided by the ratio at diagnosis. The applied statistical test was Non-parametric One sample Wilcoxon signed rank test with theoretical median = 1.0. **Supplemental Table 8C-D** presents details regarding samples included for generating the results presented in (F-G). D, diagnosis; R1/2, relapse 1/2; R2-P, persistent relapse 2.

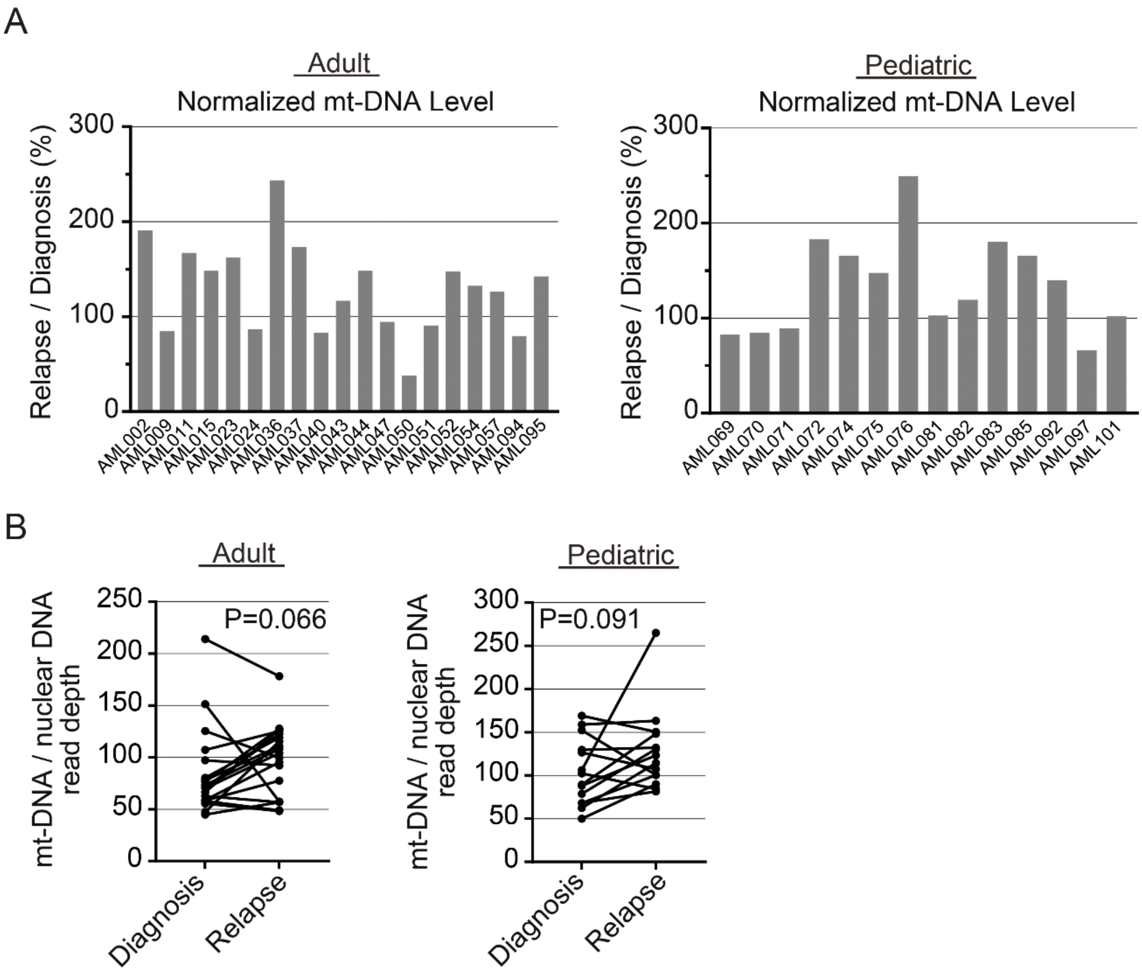

Supplemental Figure 4: Higher levels of mitochondrial DNA at AML relapse. A) Bar diagrams presenting the ratio of mitochondrial DNA read depth over the mean read depth of the nuclear genome, as presented by the ratio at relapse divided by the ratio at diagnosis on a per sample basis. **B)** Spaghetti plots showing the mitochondrial DNA amount per sample after normalization towards the mean read depth of the nuclear genome, for adults (left) and children (right). The applied statistical test was Wilcoxon matched-pairs signed rank test. **Supplemental Table 8C-D** presents details regarding samples included for generating the results presented in this figure. mt, mitochondrial.

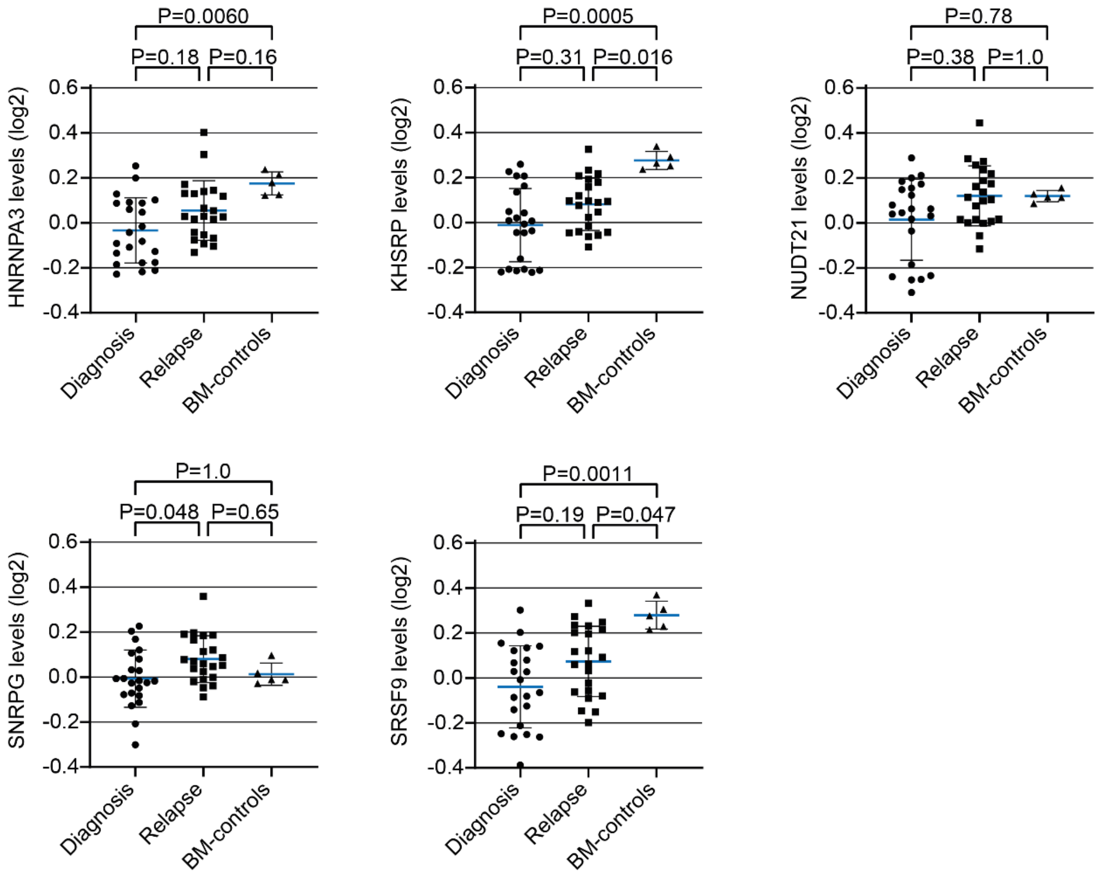

Supplemental Figure 5: The levels of splicing-related proteins differ between AML diagnosis and relapse. Scatter plots with mean (blue) and SD presenting the protein levels (log2-transformed) in adult AML samples for the five splicing-related proteins HNRNPA3, KHSRP, NUDT21, SNRPG and SRSF9 for diagnosis, relapse and BM-control samples. Applied statistical test: Kruskal-Wallis test followed by Dunn´s correction for multi-group comparisons. Visualization and underlying statistical calculations were performed by using GraphPad v.9.1.2. **Supplemental Table 8A** presents details regarding AML samples included for generating the results presented in this figure. BM-controls, CD34-expressing bone marrow samples from healthy individuals; SD, standard deviation.

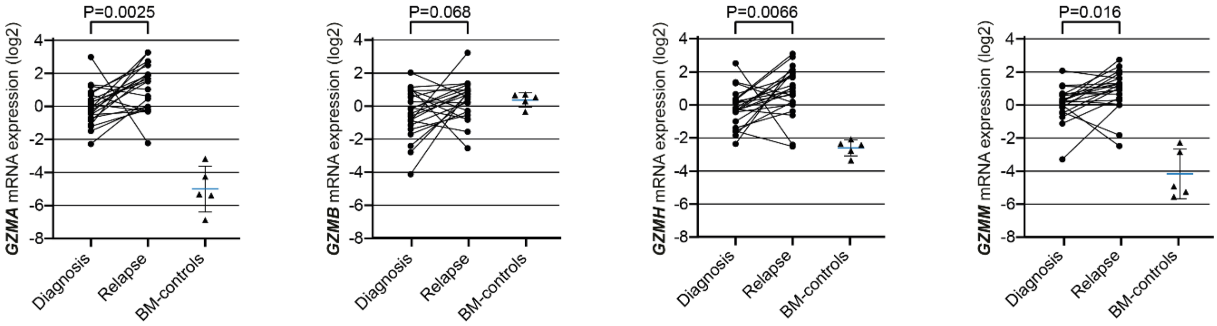

Supplemental Figure 6: Upregulation of granzymes at AML relapse is detected also at the transcriptomic level. Spaghetti plots presenting the RNA (TMM normalized and log2-transformed(5)) expression values in adult AML samples for the four granzymes *GZMA*, *GZMB*, *GZMH* and *GZMM*. Applied statistical test: Wilcoxon matched-pairs signed rank test. Each graph is overlaid with a scatter plot with mean (blue) and SD, depicting RNA expression values for the BM-controls. Visualization and underlying statistical calculations were performed by using GraphPad v.9.1.2. **Supplemental Table 8A** presents details regarding samples included for generating the results presented in this figure. BM-controls, CD34-expressing bone marrow samples from healthy individuals; SD, standard deviation; TMM, trimmed mean of M-values.

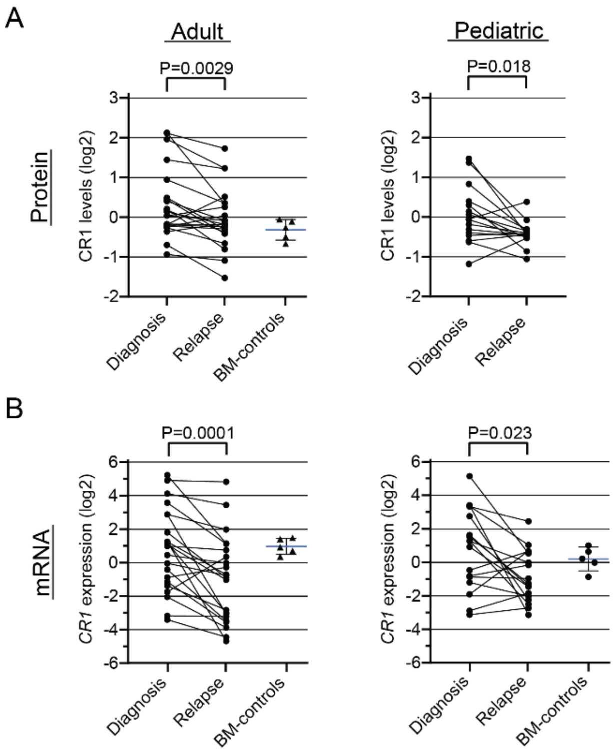

Supplemental Figure 7: Lower protein and mRNA expression of CR1 at AML relapse. A) Protein (log2 transformed) and **B**) RNA (TMM normalized and log2 transformed(5)) expression levels of CR1 in patient-matched diagnosis and relapse samples are presented in the form of spaghetti plots. Applied statistical test: Wilcoxon matched-pairs signed rank test. Each graph is overlaid with a scatter plot with mean (blue) and SD, depicting expression values for the BM-controls. No corresponding BM-control pediatric proteome data was available. Visualization and underlying statistical calculations were performed by using GraphPad v.9.2.0. **Supplemental Table 8A-B** presents details regarding AML samples included for generating the results presented in this figure. BM-controls, CD34-expressing bone marrow samples from healthy individuals; SD, standard deviation; TMM, trimmed mean of M-values.

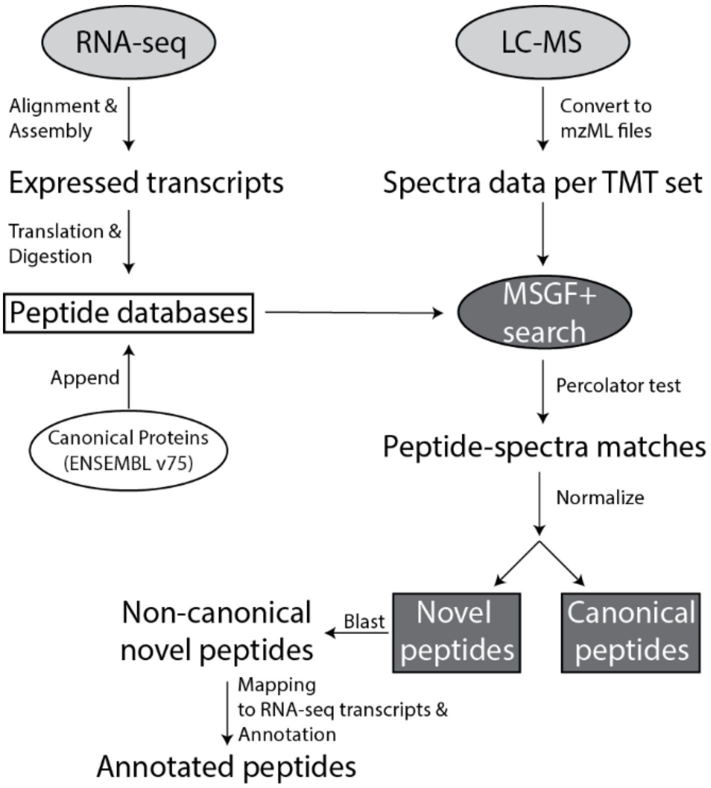

Supplemental Figure 8. Overview of the proteogenomic workflow. RNA-seq data were generated through ref.(5). A detailed description of the steps included in the proteogenomic workflow are given in the following sub-sections in the Supplemental Methods: “Sample processing and in-depth proteomics by using HiRIEF LC-MS”, “Peptide identification and quantification” and “Proteogenomic identification of novel peptides and single amino acid alterations”. LC-MS, Liquid chromatography mass spectrometry; RNA-seq, transcriptome sequencing; TMT, tandem mass tags.

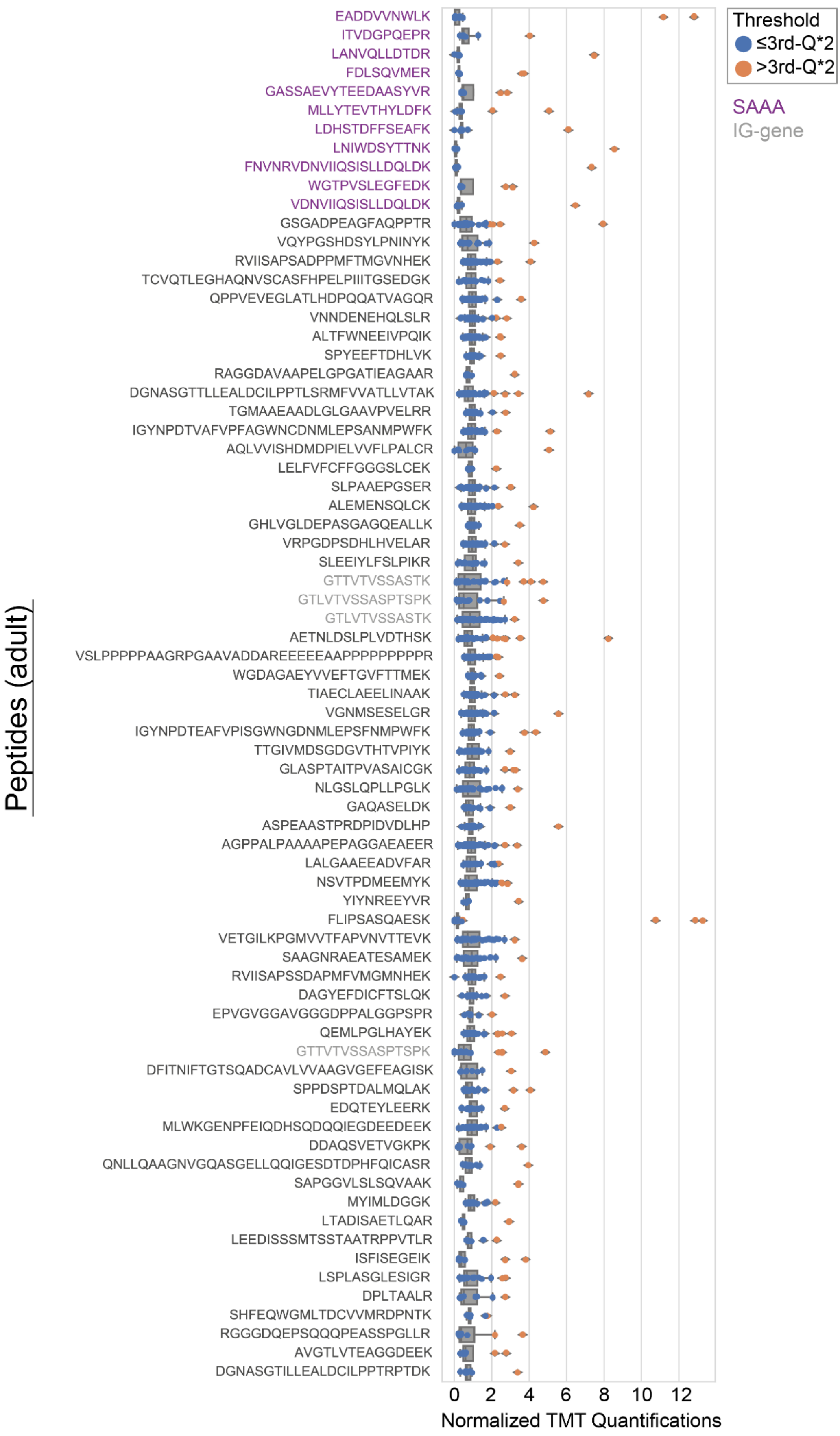
Peptides (adult)
EADDVVNWLK
ITVDGPQEPR
LANVQLLDTDR
FDLSQVMER
GASSAEVYTEEDAASYVR
MLLYTEVTHYLDFK
LDHSTDFFSEAFK
LNIWDSYTTNK
FNVNRVDNVIIQSISLLDQLDK
WGTPVSLEGFEDK
VDNVIIQSISLLDQLDK
GSGADPEAGFAQPPTR
VQYPGSHDSYLPNINYK
RVIISAPSADPPMFTMGVNHEK
TCVQTLEGHAQNVSCASFHPELPIIITGSEDGK
QPPVEVEGLATLHDPQQATVAGQR
VNNDENEHQLSLR
ALTFWNEEIVPQIK
SPYEEFTDHLVK
RAGGDAVAAPELGPGATIEAGAAR
DGNASGTTLLEALDCILPPTLSRMFVVATLLVTAK
TGMAAEAADLGLGAAVPVELRR
IGYNPDTVAFVPFAGWNCDNMLEPSANMPWFK
AQLVVISHDMDPIELVVFLPALCR
LELFVFCFFGGGSLCEK
SLPAAEPGSER
ALEMENSQLCK
GHLVGLDEPASGAGQEALLK
VRPGDPSDHLHVELAR
SLEEIYLFSLPIKR
GTTVTVSSASTK
GTLVTVSSASPTSPK
GTLVTVSSASTK
AETNLDSLPLVDTHSK
VSLPPPPPAAGRPGAAVADDAREEEEEAAPPPPPPPPPR
WGDAGAEYVVEFTGVFTTMEK
TIAECLAEELINAAK
VGNMSESELGR
IGYNPDTEAFVPISGWNGDNMLEPSFNMPWFK
TTGIVMDSGDGVTHTVPIYK
GLASPTAITPVASAICGK
NLGSLQPLLPGLK
GAQASELDK
ASPEAASTPRDPIDVDLHP
AGPPALPAAAAPEPAGGAEAEER
LALGAAEEADVFAR
NSVTPDMEEMYK
YIYNREEYVR
FLIPSASQAESK
VETGILKPGMVVTFAPVNVTTEVK
SAAGNRAEATESAMEK
RVIISAPSSDAPMFVMGMNHEK
DAGYEFDICFTSLQK
EPVGVGGAVGGGDPPALGGPSPR
QEMLPGLHAYEK
GTTVTVSSASPTSPK
DFITNIFTGTSQADCAVLVVAAGVGEFEAGISK
SPPDSPTDALMQLAK
EDQTEYLEERK
MLWKGENPFEIQDHSQDQQIEGDEEDEEK
DDAQSVETVGKPK
QNLLQAAGNVGQASGELLQQIGESDTDPHFQICASR
SAPGGVLSLSQVAAK
MYIMLDGGK
LTADISAETLQAR
LEEDISSSMTSSTAATRPPVTLR
ISFISEGEIK
LSPLASGLESIGR
DPLTAALR
SHFEQWGMLTDCVVMRDPNTK
RGGGDQEPSQQQPEASSPGLLR
AVGTLVTEAGGDEEK
DGNASGTILLEALDCILPPTRPTDK
0
2
4
6
8
10
12
Normalized TMT Quantifications
Threshold
≤3rd-Q*2
>3rd-Q*2
SAAA
IG-gene

Supplemental Figure 9: Highly abundant novel peptides in adult R/PR AML. Box plots showing the normalized quantification level per novel peptide sequence across all adult samples (including BM-controls) where the peptide is detected. Orange dots denote samples that pass the outlier threshold (third-quantile*2). Peptides derived from single amino acid alterations (SAAAs) are highlighted in purple. Peptides annotated to immunoglobulin genes (IG-genes) are marked in light gray. **Supplemental Table 8E** presents details regarding samples included for generating the results presented in this figure and **Supplemental Table 12** presents a detailed list including all novel peptides. Q, quantile; R/PR AML, relapse and primary resistant acute myeloid leukemia; TMT, tandem mass tags.

557 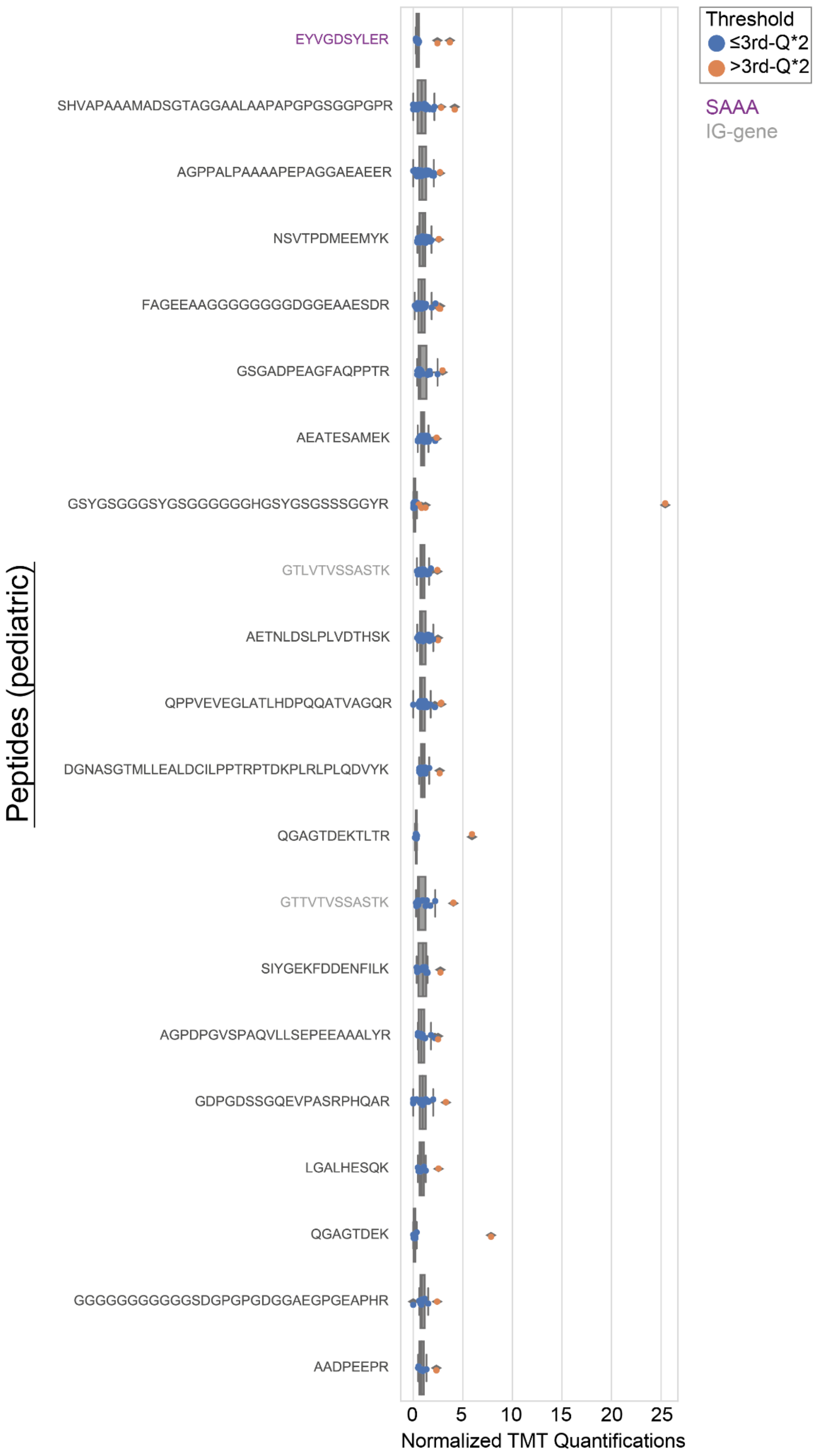

EYVGDSYLER
SHVAPAAAMADSGTAGGAALAAPAPGPGSGGPGPR
AGPPALPAAAAPEPAGGAEAEER
NSVTPDMEEMYK
FAGEEAAGGGGGGGGDGGEAAESDR
GSGADPEAGFAQPPTR
AEATESAMEK
GSYGSGGGSYGSGGGGGGHGSYGSGSSSGGYR
GTLVTVSSASTK
AETNLDSLPLVDTHSK
QPPVEVEGLATLHDPQQATVAGQR
DGNASGTMLLEALDCILPPTRPTDKPLRLPLQDVYK
QGAGTDEKTLTR
GTTVTVSSASTK
SIYGEKFDDENFILK
AGPDPGVSPAQVLLSEPEEAAALYR
GDPGDSSGQEVPASRPHQAR
LGALHESQK
QGAGTDEK
GGGGGGGGGGGSDGPGPGDGGAEGPGEAPHR
AADPEEPR
Peptides (pediatric)
0
5
10
15
20
25
Normalized TMT Quantifications
Threshold
≤3rd-Q*2
>3rd-Q*2
SAAA
IG-gene

Supplemental Figure 10: Highly abundant novel peptides in pediatric R/PR AML. Box plots showing the normalized quantification level per novel peptide sequence across all pediatric samples where the peptide is detected. Orange dots denote samples that pass the outlier threshold (third-quantile*2). A peptide derived from a single amino acid alteration (SAAA) is highlighted in purple. Peptides annotated to immunoglobulin genes (IG-genes) are marked in light gray. **Supplemental Table 8F** presents details regarding samples included for generating the results presented in this figure and **Supplemental Table 13** presents a detailed list including all novel peptides. Q, quantile, R/PR AML, relapse and primary resistant acute myeloid leukemia; TMT tandem mass tags.

SUPPLEMENTAL REFERENCES

1. Glimelius B, Melin B, Enblad G, Alafuzoff I, Beskow A, Ahlstrom H, et al. U-CAN: a prospective longitudinal collection of biomaterials and clinical information from adult cancer patients in Sweden. Acta Oncol. 2018;57(2):187-94.
2. Arber DA, Orazi A, Hasserjian R, Thiele J, Borowitz MJ, Le Beau MM, et al. The 2016 revision to the World Health Organization classification of myeloid neoplasms and acute leukemia. Blood. 2016;127(20):2391-405.
3. Dohner H, Estey E, Grimwade D, Amadori S, Appelbaum FR, Buchner T, et al. Diagnosis and management of AML in adults: 2017 ELN recommendations from an international expert panel. Blood. 2017;129(4):424-47.
4. Stratmann S, Yones SA, Mayrhofer M, Norgren N, Skaftason A, Sun J, et al. Genomic characterization of relapsed acute myeloid leukemia reveals novel putative therapeutic targets. Blood Adv. 2021;5(3):900-12.
5. Stratmann S, Yones SA, Garbulowski M, Sun J, Skaftason A, Mayrhofer M, et al. Transcriptomic analysis reveals proinflammatory signatures associated with acute myeloid leukemia progression. Blood Adv. 2022;6(1):152-64.
6. Moggridge S, Sorensen PH, Morin GB, Hughes CS. Extending the Compatibility of the SP3 Paramagnetic Bead Processing Approach for Proteomics. Journal of Proteome Research. 2018;17(4):1730-40.
7. Hughes CS, Foehr S, Garfield DA, Furlong EE, Steinmetz LM, Krijgsveld J. Ultrasensitive proteome analysis using paramagnetic bead technology. Molecular systems biology. 2014;10(10):757.
8. Branca RM, Orre LM, Johansson HJ, Granholm V, Huss M, Perez-Bercoff A, et al. HiRIEF LC-MS enables deep proteome coverage and unbiased proteogenomics. Nat Methods. 2014;11(1):59-62.
9. Holman JD, Tabb DL, Mallick P. Employing ProteoWizard to Convert Raw Mass Spectrometry Data. Curr Protoc Bioinformatics. 2014;46:13.24.1-9.
10. Kim S, Pevzner PA. MS-GF+ makes progress towards a universal database search tool for proteomics. Nature Communications. 2014;5(1):5277.
11. Granholm V, Kim S, Navarro JCF, Sjölund E, Smith RD, Käll L. Fast and Accurate Database Searches with MS-GF+Percolator. Journal of Proteome Research. 2014;13(2):890-7.
12. Boekel J, Chilton JM, Cooke IR, Horvatovich PL, Jagtap PD, Käll L, et al. Multi-omic data analysis using Galaxy. Nat Biotechnol. 2015;33(2):137-9.
13. Boekel J. Nextflow ddamsproteomics github [updated 2020-09-25. Available from: https://github.com/lehtiolab/ddamsproteomics#readme.
14. Röst HL, Sachsenberg T, Aiche S, Bielow C, Weisser H, Aicheler F, et al. OpenMS: a flexible open-source software platform for mass spectrometry data analysis. Nat Methods. 2016;13(9):741-8.
15. Savitski MM, Wilhelm M, Hahne H, Kuster B, Bantscheff M. A Scalable Approach for Protein False Discovery Rate Estimation in Large Proteomic Data Sets. Molecular & cellular proteomics : MCP. 2015;14(9):2394-404.
16. Pearson K. LIII. On lines and planes of closest fit to systems of points in space. The London, Edinburgh, and Dublin Philosophical Magazine and Journal of Science. 1901;2(11):559-72.
17. Hotelling H. Analysis of a complex of statistical variables into principal components. Journal of Educational Psychology. 1933;24(6):417-41.
18. Jolliffe IT. Principal component analysis. New-York: Springer-Verlag; 2002.

19. Van der Maaten L, Hinton G. Visualizing data using t-SNE. Journal of machine learning research. 2008;9(11).
20. Van Der Maaten L. Accelerating t-SNE using tree-based algorithms. The Journal of Machine Learning Research. 2014;15(1):3221-45.
21. Eisen MB, Spellman PT, Brown PO, Botstein D. Cluster analysis and display of genome-wide expression patterns. Proceedings of the National Academy of Sciences. 1998;95(25):14863-8.
22. Benjamini Y, Hochberg Y. Controlling the false discovery rate: a practical and powerful approach to multiple testing. J R Stat Soc B. 1995;57:289-300.
23. Eden E, Lipson D, Yogev S, Yakhini Z. Discovering Motifs in Ranked Lists of DNA Sequences. PLOS Computational Biology. 2007;3(3):e39.
24. Eden E, Navon R, Steinfeld I, Lipson D, Yakhini Z. GOrilla: a tool for discovery and visualization of enriched GO terms in ranked gene lists. BMC Bioinformatics. 2009;10(1):48.
25. Kovaka S, Zimin AV, Pertea GM, Razaghi R, Salzberg SL, Pertea M. Transcriptome assembly from long-read RNA-seq alignments with StringTie2. Genome Biology. 2019;20(1):278.
26. Cock PJ, Antao T, Chang JT, Chapman BA, Cox CJ, Dalke A, et al. Biopython: freely available Python tools for computational molecular biology and bioinformatics. Bioinformatics. 2009;25(11):1422-3.
27. Consortium TU. UniProt: a worldwide hub of protein knowledge. Nucleic Acids Res. 2018;47(D1):D506-D15.
28. Frankish A, Diekhans M, Ferreira AM, Johnson R, Jungreis I, Loveland J, et al. GENCODE reference annotation for the human and mouse genomes. Nucleic Acids Res. 2019;47(D1):D766-d73.
29. McLaren W, Gil L, Hunt SE, Riat HS, Ritchie GR, Thormann A, et al. The Ensembl Variant Effect Predictor. Genome Biol. 2016;17(1):122.
30. Zhu Y, Orre LM, Johansson HJ, Huss M, Boekel J, Vesterlund M, et al. Discovery of coding regions in the human genome by integrated proteogenomics analysis workflow. Nat Commun. 2018;9(1):903.
31. Kent WJ, Sugnet CW, Furey TS, Roskin KM, Pringle TH, Zahler AM, et al. The human genome browser at UCSC. Genome Res. 2002;12(6):996-1006.
32. Aasebø E, Berven FS, Hovland R, Døskeland SO, Bruserud Ø, Selheim F, et al. The Progression of Acute Myeloid Leukemia from First Diagnosis to Chemoresistant Relapse: A Comparison of Proteomic and Phosphoproteomic Profiles. Cancers (Basel). 2020;12(6).
